# Investigation of Oxidative-Stress Impact on Human Osteoblasts During Orthodontic Tooth Movement Using an In Vitro Tension Model

**DOI:** 10.3390/ijms252413525

**Published:** 2024-12-17

**Authors:** Samira Hosseini, Julia Diegelmann, Matthias Folwaczny, Hisham Sabbagh, Sven Otto, Tamara Katharina Kakoschke, Andrea Wichelhaus, Uwe Baumert, Mila Janjic Rankovic

**Affiliations:** 1 Department of Orthodontics and Dentofacial Orthopedics, LMU University Hospital, LMU Munich, 80336 Munich, Germany; samirahosseini1251@gmail.com (S.H.); hisham.sabbagh@med.uni-muenchen.de (H.S.); kfo.sekretariat@med.uni-muenchen.de (A.W.); uwe.baumert@med.uni-muenchen.de (U.B.); 2 Department of Conservative Dentistry and Periodontology, LMU University Hospital, LMU Munich, 80336 Munich, Germany; julia.diegelmann@med.uni-muenchen.de (J.D.); matthias.folwaczny@med.uni-muenchen.de (M.F.); 3 Department of Oral and Maxillofacial Surgery and Facial Plastic Surgery, LMU University Hospital, LMU Munich, 80337 Munich, Germany; sven.otto@med.uni-muenchen.de (S.O.); tamara.kakoschke@med.uni-muenchen.de (T.K.K.)

**Keywords:** human osteoblasts, tensile strain, oxidative stress, bone remodeling, orthodontic tooth movement

## Abstract

In recent years, there has been a growing number of adult orthodontic patients with periodontal disease. The progression of periodontal disease is well-linked to oxidative stress (OS). Nevertheless, the impact of OS on orthodontic tooth movement (OTM) is not fully clarified. Therefore, we applied an OS in vitro-model utilizing H_2_O_2_ to study its effect on tension-induced mechanotransduction in human osteoblasts (hOBs). Experimental parameters were established based on cell viability and proliferation. Apoptosis detection was based on caspase-3/7 activity. Gene expression related to bone-remodeling (*RUNX2*, *P2RX7*, *TNFRSF11B*/*OPG*), inflammation (*CXCL8*/*IL8*, *IL6*, *PTRGS2*/*COX2*), autophagy (*MAP1LC3A*/*LC3*, *BECN1*), and apoptosis (*CASP3*, *CASP8*) was analyzed by RT-qPCR. IL6 and PGE2 secretion were determined by ELISA. Tension increased the expression of *PTRGS2*/*COX2* in all groups, especially after stimulation with higher H_2_O_2_ concentration. This corresponds also to the measured PGE2 concentrations. *CXCL8*/*IL8* was upregulated in all groups. Cells subjected to tension alone showed a general upregulation of osteogenic differentiation-related genes; however, pre-stimulation with OS did not induce significant changes especially towards downregulation. *MAP1LC3A*/*LC3*, *BECN1* and *CASP8* were generally upregulated in cells without OS pre-stimulation. Our results suggest that OS might have considerable impacts on cellular behavior during OTM.

## 1. Introduction

Orthodontic tooth movement (OTM) serves as a therapeutic approach to correct misaligned and/or dispositioned teeth. OTM involves mechanical stimulation, eliciting intricate aseptic inflammatory cellular and molecular responses that lead to tissue remodeling. This results in bone resorption on the compression side and bone apposition on the tension side [1]. In recent years, there has been a notable increase in the number of adult orthodontic patients with preexisting periodontal disease [2]. It is well-established that both orthodontic force and periodontal disease exert a significant influence on cellular and tissue homeostasis [3,4]. They stimulate bone remodeling, inflammatory responses, and pivotal biological processes such as autophagy and apoptosis [5]. Like various inflammatory diseases, periodontal disease is associated with oxidative stress (OS)—a condition arising from an imbalance between the production and accumulation of reactive oxygen species (ROS) in cells and tissues [6,7,8]. ROS, including hydrogen peroxide (H_2_O_2_), are highly reactive oxygen metabolites produced in living organisms as a by-product of aerobic processes [9,10]. Under normal conditions, antioxidants neutralize ROS, preventing tissue damage. However, during inflammation, ROS production escalates, overloading the antioxidant defense system and leading to oxidative stress and tissue damage [6,11]. ROS can directly induce tissue damage through lipid peroxidation, DNA damage, protein damage, and enzyme oxidation [6]. Additionally, they serve as important signaling molecules or mediators of inflammation [6], indirectly influencing the production of cytokines, chemokines, and enzymes, interplaying with multiple pathways. For example, oxidative stress can trigger the expression of *IL6*, *CXCL8/IL8*, and *PTGS2/COX2*, which are involved in inflammatory responses and immune modulation [12]. As a signaling factor, ROS are known to influence bone metabolism by affecting osteoblasts. Consequently, a reduced osteoblastogenesis and a disruption in normal cellular signaling pathways might occur, leading to an imbalance between bone formation and resorption [13].

Clinically, orthodontic treatment should be initiated after periodontal therapy during remission [4,14]. This is also linked to improved redox balance and a reduction in inflammatory parameters, mitigating the damaging effects of oxidative stress on cellular function, but not completely restoring it [15,16]. While increased oxidative stress is a hallmark of periodontal disease and is proven to influence many molecular events including bone remodeling, inflammation and cell destiny [7], its effect on OTM is not fully clarified. Therefore, this study aims to address this gap by investigating the interplay between OS and mechanosensitive gene expression in human alveolar osteoblasts (hOBs) under combined conditions of oxidative and mechanical stress.

**Figure 1:**
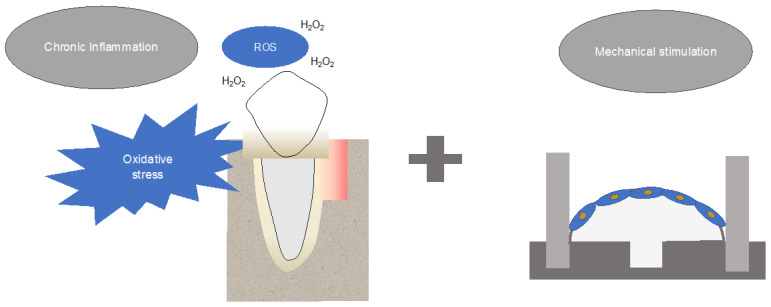
Oxidative stress in vitro-model utilizing H_2_O_2_ [21] and tensile strain [20] to study its effect on mechanotransduction in primary human alveolar osteoblasts.

To establish an OTM-related OS in vitro model, two well-established in vitro setups were combined. The effect of OS was simulated by exposing cells to H_2_O_2_ [17,18]. Afterwards, the cells were mechanically stimulated using tensile strain [19,20] (Figure 1). Suitable experimental parameters were established based on cell viability and proliferation. Gene expression related to bone remodeling (*RUNX2*, *P2RX7*, *TNFRSF11B*/*OPG*), inflammation (*IL6*, *CXCL8/IL8*, *PTGS2/COX2*), autophagy (*MAP1LC3A*/*LC3*, *BECN1*) and apoptosis (*CASP3*, *CASP8*) was examined.

## 2. Results

### 2.1. Hydrogen Peroxide (H_2_O_2_) Concentration Testing

To identify the lowest concentrations causing a cytotoxic effect without affecting cell viability, different H_2_O_2_ concentrations ranging from 20 µM to 500 µM were tested on primary hOBs. Unstimulated (i.e., 0 µM H_2_O_2_) but otherwise identically treated cells served as controls.

**Figure 2:**
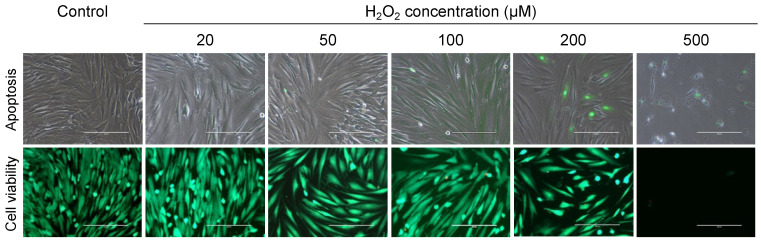
Effects of different hydrogen peroxide concentrations ranging from 0 µM (i.e., control) to 500 µM on apoptosis induction (**upper row**) and cell viability (**lower row**) on primary human osteoblasts. **Upper row:** Apoptosis induction was detected using the CellEvent™ Caspase-3/7 Detection Reagent (R37111; Life Technologies, Carlsbad, CA, USA). Caspase-3/7-positive cells were stained green (overlay of fluorescence and phase contrast). **Lower row:** Cell viability assessment using live/dead cell staining. Green cells represent living cells. Dead cells are either detached and washed away or stained with the red color. (Scale: 200 µm).

Apoptosis induction and cell viability were assessed qualitatively by caspase-3/7 activity detection using a specific reagent (R37111; Life Technologies, Carlsbad, CA, USA) and a live/dead cell staining kit (L3224; ThermoFisher Scientific, Carlsbad, CA, USA), respectively (Figure 2). The higher the concentration of H₂O₂ applied, the fewer living cells were observed, while caspase-3/7 positive cells became more prominent. These changes were especially visible in groups stimulated with 200 µM and 500 µM of H_2_O_2_.

**Figure 3:**
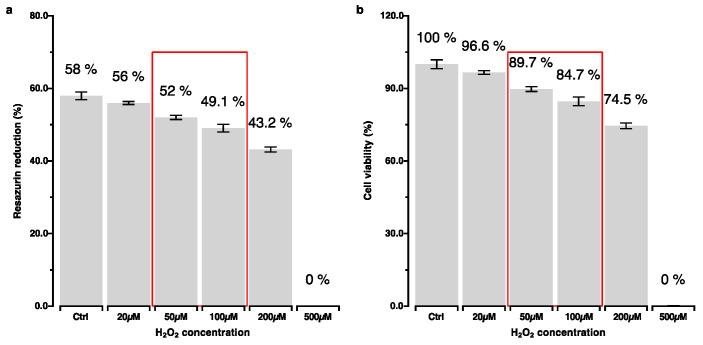
Percentage reduction of resazurin: (**a**) Cytotoxic effect of H_2_O_2_, (**b**) cell viability calculated as normalized resazurin reduction relative to the control group. 50 µM and 100 µM were identified as the lowest concentrations of H_2_O_2_ showing a cytotoxic effect; however, these did not have pronounced effects on cell viability.

The impacts of the different H_2_O_2_ concentrations on cytotoxicity and cell viability were quantitatively determined using a resazurin-based assay (Figure 3). The findings are in line with the results of the apoptosis and cell viability tests (Figure 2).

Based on these results, H_2_O_2_ concentrations of 50 µM and 100 µM were chosen to further investigate the effects of higher and lower OS exposure levels.

### 2.2. Effect of Oxidative Stress Induction Alone on Gene Expression Immediately After H₂O₂ Incubation and After Additional 24 h Post-Incubation

**Figure 4:**
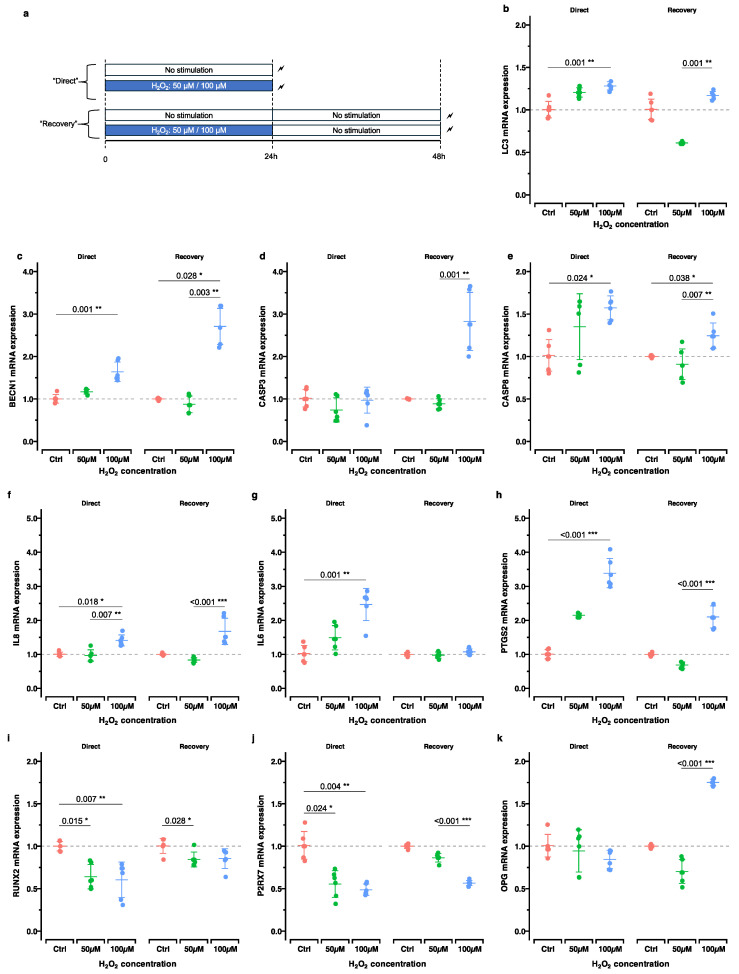
Effect of oxidative stress induction alone on gene expression immediately after H₂O₂ incubation (“direct”) and 24 h post-incubation (“recovery”). (**a**) Experimental design. (**b**–**k**) RT-qPCR results for genes related to autophagy (**b**,**c**, *MAP1LC3A*/*LC3*, *BECN1*), apoptosis (**d**,**e**, *CASP3*, *CASP8*), inflammation (**f**–**h**, *CXCL2/IL8*, *IL6*, *PTGS2/COX2*), and bone remodeling (**i**–**k**, *RUNX2*, *P2RX7*, *TNFRSF11B*/*OPG*). For each genetic locus, gene expression directly after H_2_O_2_ exposure (left panel, “direct”) and after an additional 24 h cultivation in H_2_O_2_-free cell culture medium (right panel, “recovery”) is depicted. Adjusted *p*-values based on multiple comparisons within each experimental group are reported: *, *p*_adj_. < 0.05; **, *p*_adj_. < 0.01; ***, *p*_adj_. < 0.001.

Herein, we evaluated the immediate and lasting effects of oxidative stress induction on gene expression. hOBs were stimulated with either 50 µM or 100 µM H_2_O_2_ for 24 h. The expressions of genes related to bone remodeling, inflammation, autophagy and apoptosis were determined either immediately after the stimulation (“direct”) or after an additional 24 h post-incubation period in H_2_O_2_-free medium (“recovery”) (Figure 4, Table 1).

The autophagy or apoptosis-related genes *MAP1LC3A*/*LC3*, *BECN1* and *CASP8* showed dose-dependent upregulation patterns immediately after H_2_O_2_ stimulation. Similar gene expression regulation patterns were observed after 24 h post-incubation concerning the apoptosis-related gene *CASP3* (*p*_adj_. = 0.001) and the autophagy-related gene *BECN1* (*p*_adj_. = 0.028) stimulated with 100 µM H_2_O_2_ (Figure 4b–e).

In all three inflammation-related genes, *CXCL8*/*IL8*, *IL6* and *PTGS2*/*COX2*, a concentration-dependent upregulated expression was observed directly after H_2_O_2_ stimulation (Figure 4f–h). After 24 h post-incubation, a recovery effect was observable. While *IL6* gene expression was downregulated to control level, pre-stimulation with 100 µM H_2_O_2_ led to a persistent upregulation of *CXCL8*/*IL8* and *PTGS2*/*COX2* after 24 h post-incubation (*CXCL8*/*IL8*—*p*_adj_. < 0.001; *PTGS2/COX2*—*p*_adj_. < 0.001).

The genes related to bone remodeling were concentration-dependently downregulated directly after H_2_O_2_ stimulation depending on the concentration (Figure 4i–k). After 24 h post-incubation, a recovery of *RUNX2* and *P2RX7* expression was observed, but still below the corresponding controls. This contrasted with *TNFRSF11B*/*OPG*, which was downregulated directly after H_2_O_2_ stimulation, showing partially contradicting results 24 h post-exposure; 50 µM H_2_O_2_ resulted in a more pronounced downregulation, whereas 100 µM H_2_O_2_ led to an upregulation compared to the control (mean FC: 1.75).

### 2.3. Expression of Genes and Metabolites Related to Inflammation, Bone Remodeling, Apoptosis and Autophagy in Mechanically Stimulated Cells with and Without Previous OS Stimulation

**Figure 5:**
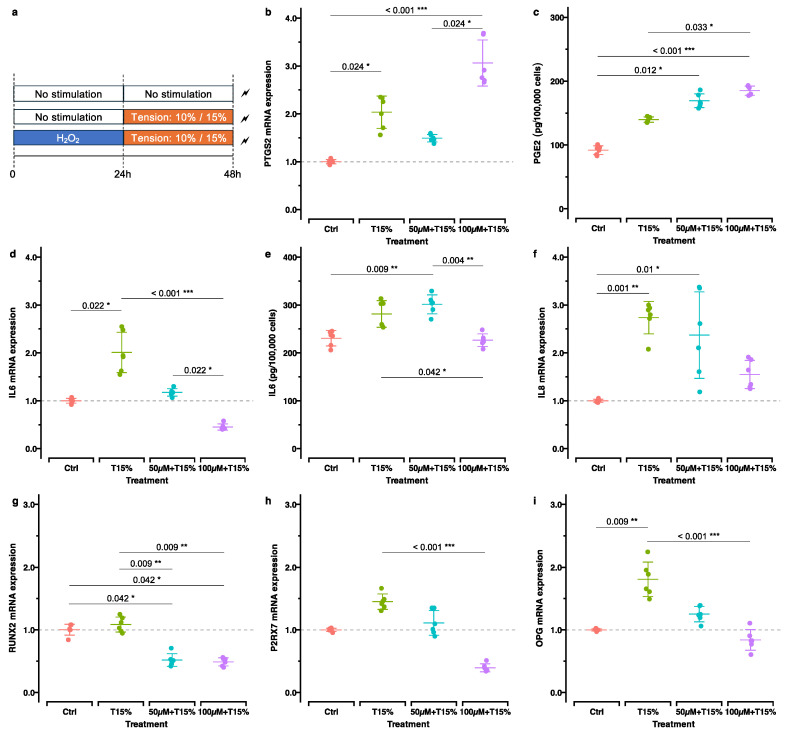
Expressions of genes and metabolites related to inflammation and bone remodeling in mechanically stimulated cells with and without previous H_2_O_2_ stimulation. (**a**) Experimental setup: the control group (ctrl) received neither H_2_O_2_ nor tension stimulation. The tension (T10%, T15%) group was stimulated by static tension after 24 h non-stimulation. The H_2_O_2_/tension group was stimulated for 24 h with 50 µM or 100 µM H_2_O_2_ followed by 24 h static tension at 10% or 15% stretching. Shown here are the results from 15% tension stimulation. (**b**–**f**) The expression of inflammation-related genes and metabolites and (**g**–**i**) genes related to bone remodeling are reported. Shown are the adjusted *p*-values based on multiple comparisons between each experimental treatment (*, *p*_adj_. < 0.05; **, *p*_adj_. < 0.01; ***, *p*_adj_. < 0.001). Results derived from 10% tension are reported in Supplementary Materials S1.

Next, we investigated the effects of the static tension on hOBs with and without previous H_2_O_2_ stimulation focusing on the gene expression related to inflammation, bone remodeling, apoptosis and autophagy (Figure 5a, Table 2). Based on a previously published review [22], our initial intention was to test two different tensile strains—10% as the most frequently used one in studies applying static tension, and 15% to simulate a more tensile strains. However, due to almost identical gene/protein expression patterns, we decided to present only results derived from the 15% tensile strain setup. Nevertheless, results derived from the 10% tension setup are summarized in Supplementary Materials S1.

#### 2.3.1. Inflammation

After tension force application, *IL6* expression was significantly more strongly upregulated in cells without prior OS induction than in the groups with OS preinduction, especially in the experimental group stimulated with 100 µM H_2_O_2_ (*p*_adj_. < 0.001). This was also confirmed by ELISA (*p*_adj_. = 0.042). Tension force also increased the expression of the inflammatory gene *PTGS2*/*COX2* in all groups, especially in the group previously stimulated with 100 µM H_2_O_2_ concentration (*p*_adj_. < 0.001). These results correspond to the measured PGE2 concentrations in supernatants reflecting *PTGS2*/*COX2* activity. *CXCL8*/*IL8* was also upregulated in all groups, with the highest upregulation in cells without previous OS induction (Figure 5b–f).

#### 2.3.2. Bone Remodeling

The general stimulatory effect of tension force on genes related to bone formation was observed in groups of cells without H_2_O_2_ pre-stimulation (Figure 5g–i). This effect was either less pronounced or even caused downregulation in groups previously subjected to H_2_O_2_ stimulation compared to control.

#### 2.3.3. Apoptosis and Autophagy

**Figure 6:**
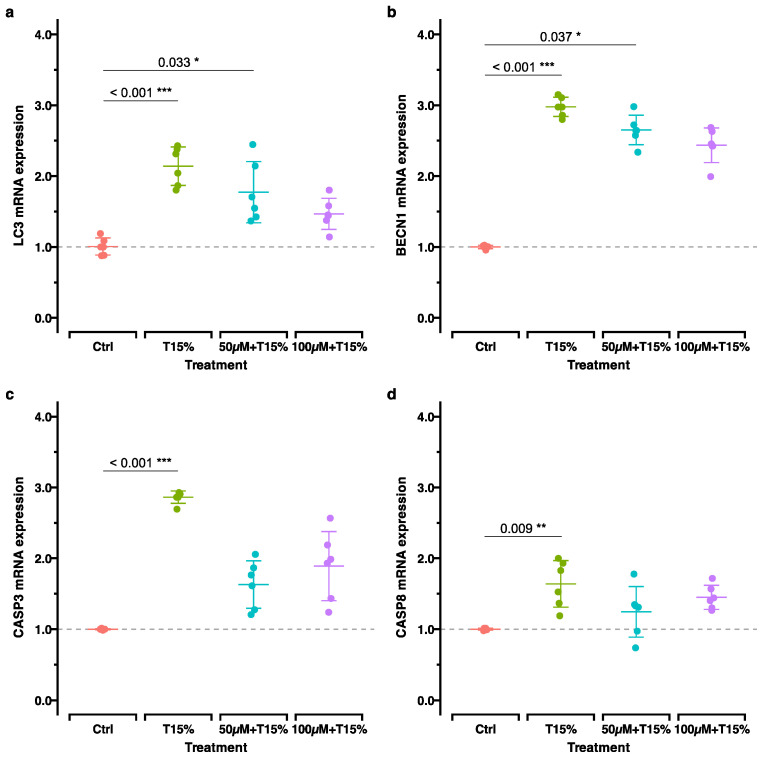
RT-qPCR results for autophagy- (**a**,**b**) and apoptosis (**c**,**d**)-related genes. Shown are the adjusted *p*-values based on multiple comparisons between each experimental treatment. The groups are the same as in Figure 5. *, *p*_adj_. < 0.05; **, *p*_adj_. < 0.01; ***, *p*_adj_. < 0.001.

Apoptosis- and autophagy-related genes were upregulated in all experimental groups compared to the control; however, this was considerably stronger in cells without previous H_2_O_2_ stimulation (Figure 6).

#### 2.3.4. Effect of Static Tension on Viability and Proliferation

**Figure 7:**
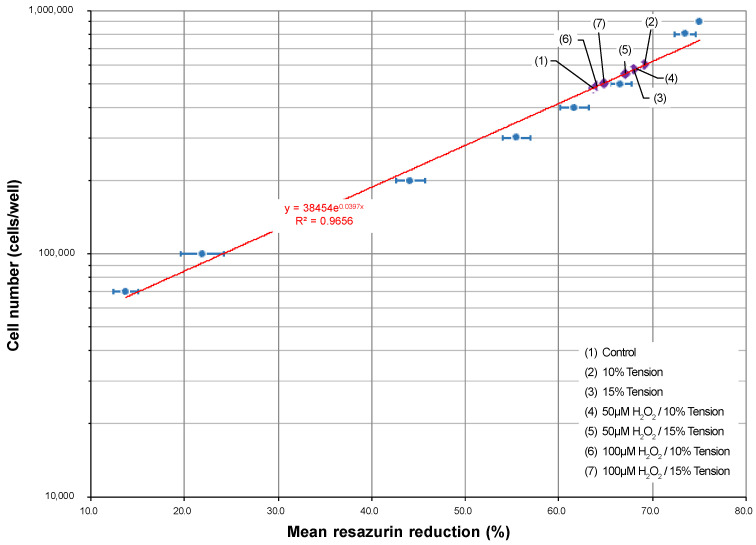
Establishment of a standard curve to assess cell growth of the hOBs used in the experiments. The resazurin standard curve was prepared as described in materials and methods. hOBs of the 5th passage were seeded in triplicate (70,000; 100,000; 200,000; 300,000; 400,000; 500,000) or duplicate (800,000 and 900,000 cells per well). Exponential regression was used to calculate the standard curve (red line) (Microsoft Excel). The cellular growth of hOBs in the different experimental setups (legend: lower right) is shown with violet diamonds (♦) on the fitted curve.

**Figure 8:**
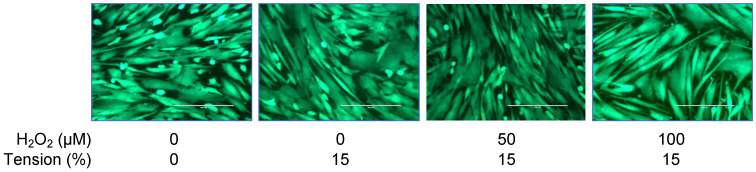
Qualitative assessment of cell viability of cells belonging to the different experimental groups using live/dead cell staining. Independent of the experimental group, cells proved to be viable (green staining). Dead cells were rarely observed (red staining). (Scale bar: 200 μm). (Data from experiments with 10% tension are provided in Supplementary Materials S1.

To estimate the cell number, a standard curve was established based on resazurin reduction (Figure 7). Generally, tensile strain seemed to have a positive effect on proliferation; however, the groups previously exposed to H_2_O_2_ showed a slower proliferation tendency. This is also in line with live/dead staining results (Figure 8).

## 3. Discussion

With the rising number of adult patients seeking orthodontic treatment in recent years, orthodontists are more frequently encountering individuals with periodontal disease [2]. The dysregulation of redox homeostasis under pathological conditions characterized by chronic inflammation, such as periodontal disease, results in the excessive generation of reactive oxygen species (ROS), leading to oxidative stress (OS). Although it is known that OS has an important influence on processes that are also affected by mechanical stimulation, such as inflammation, bone metabolism, autophagy and apoptosis, the role of OS in relation to orthodontic tooth movement (OTM) is still largely unknown.

Therefore, this in vitro study aimed to elucidate this topic focusing on cells centrally involved in OTM, human osteoblasts derived from the alveolar bone, and static tension force, as one of the most dominant forces during OTM. For the simulation of oxidative stress, we applied H_2_O_2_ as an ROS stimulus.

### 3.1. Experimental Parameters and Viability and Proliferation Assessment in Relation to OS Stimulation

There are several ways to experimentally study OS in cell cultures, where OS is induced by exposure to various chemicals that promote ROS generation, such as menadione, paraquat, and potassium bromate, or by exposure to ROS directly, such as H₂O₂, superoxide anion, hydroxyl radical, and peroxynitrite [23]. Menadione primarily generates superoxide and has been widely used to study ROS-related cell damage and proliferation [23,24]. Potassium bromate induces oxidative stress through distinct pathways, while other oxidants, such as hydroxyl radicals and peroxynitrite, have been employed to study specific cellular effects, including apoptosis and mitochondrial dysfunction [23,25,26]. H₂O₂ is both an ROS and an inducer of further ROS formation, playing a dual role in oxidative stress and cellular signaling. Its use as a stressor is well-established across various cell types, including osteoblasts [27] and periodontal ligament cells [28,29,30,31]. The widespread application of H₂O₂ in experimental setups and its reliability as a model for simulating oxidative stress were key factors in our decision to use it in this study.

Although H_2_O_2_ is widely used in in vitro experiments, its cytotoxicity varies between different cell cultures and thus has to be individually defined for different experimental cell models [32]. Therefore, in all experimental procedures applied in this study, special attention was paid to monitoring cell viability and proliferation. Herein, the experimental parameters were chosen to identify the lowest concentrations of H_2_O_2_ showing a relevant cytotoxic effect, but without pronounced negative effects on cell viability. H_2_O_2_ is a non-radical ROS, lacking an unpaired electron, and thus exhibits moderate reactivity [33]. The ability of H_2_O_2_ to easily penetrate membranes, to migrate significant distances from its production site, and to maintain high stability, enables it to exert its effects at various cellular locations [34]. Physiologically, H_2_O_2_ demonstrates dual roles in cells, both as a toxic agent and a signaling molecule critical for cellular defense and regulation [34,35]. This dual role is concentration-dependent and exhibits a biphasic effect. At low concentrations, H₂O₂ acts as a signaling molecule that supports cellular proliferation, differentiation, and survival [35]. It achieves this by modulating intracellular redox status, upregulating glutathione, and activating DNA-binding proteins like those targeting the antioxidant response element. These actions contribute to maintaining cellular homeostasis and enhancing adaptive responses to mild oxidative stress [11,35,36]. In contrast, at high concentrations, H_2_O_2_ is known to have negative effects on cell proliferation, and inflict severe cellular damage through oxidative modifications of proteins and DNA, leading to cell death [33,37].

Clinically, this dual role could have significant implications. Under inflammatory conditions or during aging, where dysregulated ROS levels exacerbate tissue damage, understanding and harnessing the dose-dependent effects of H₂O₂ could guide the development of antioxidant-based treatments to restore redox balance without disrupting essential signaling pathways.

OTM induces differential responses in osteoblast activity, with the stimulation of tissue formation expected on the tension side [38]. According to a recent systematic review [39], tension signals can increase the proliferation in hOBs. In line with these findings, we observed stronger proliferation in cells subjected to tension. However, this was less pronounced if the cells were previously exposed to higher H_2_O_2_ concentrations, suggesting a potential inhibiting influence of OS on cell proliferation during mechanical stimulation with tensile strain. These results indicate that oxidative stress may affect orthodontic treatment outcomes in periodontal patients, as an impaired proliferation of osteoblasts could limit the desired response to mechanical forces.

### 3.2. Gene and Protein Expression Related to Inflammation

Herein, we investigate the expression of three different regulatory genes known to have a critical role in the regulation of inflammation: *IL6*, *CXCL8*/*IL8*, and *PTGS2*/*COX2*. The latter is an enzyme that converts arachidonic acid into prostaglandins, including PGE2. It has been shown that both PGE2 and *PTGS2*/*COX2* regulate inflammation-associated processes by modulating the secretion of cytokines. PGE2 can enhance the synthesis of *IL6*, a pro-inflammatory cytokine, which, if not properly controlled, may result in chronic inflammation and the progression of many inflammatory diseases, including various oral entities, i.e., periodontitis [40,41]. Similarly, PGE2 can also promote the synthesis of *CXCL8/IL8*, which is crucial for attracting neutrophils to sites of infection or inflammation [42]. These cytokines are also essential for coordinating the resorption and formation of bone during OTM, ensuring that the teeth can be moved effectively [43].

It is generally accepted that H_2_O_2_ at micromolar levels can function as a second messenger, initiating inflammatory responses [44]. Gene expression in hOBs cell culture directly after H_2_O_2_ incubation showed dose-dependent increases in the proinflammatory gene expressions of all three genes. Although less expressed, this proinflammatory effect was still observable in cells treated with higher H_2_O_2_ concentrations after 24 h post-incubation. On the contrary, in cells exposed to lower H_2_O_2_ concentrations, proinflammatory markers were found to be non- or downregulated after 24 h post-incubation. These results suggest a diminishing, but still present, dose-dependent altering effect of OS on cellular function in hOBs. Although these in vitro findings presented here are in line with clinical observations linking oxidative stress marker levels to the severity of periodontal inflammation [15,45,46,47], they should not be overinterpreted.

Despite being related to anabolic processes during OTM, like OS, tensile strain is also known to induce proinflammatory responses within the surrounding tissues [22]. This is confirmed by many in vitro studies, which were reviewed recently with special focus on human primary PDL cells [22]. However, information derived from studies using hOBs is limited [48,49], and to our knowledge, this is the first study investigating this topic in relation to OS stimulation. Based on our results, the mechanical stimulation of cells previously exposed to oxidative stress (OS) appeared to have a different effect on proinflammatory gene expression. Specifically, for genes like *IL6* and *CXCL8*/*IL8*, tension seems to induce less proinflammatory gene expression in cells previously exposed to OS compared to cells exposed to tension alone. *IL6* and *CXCL8/IL8* are central cytokines in the inflammatory response, and their dysregulation has been strongly implicated in the pathogenesis of periodontal diseases such as gingivitis and periodontitis [50,51]. In periodontitis, the upregulation of *IL6* and *CXCL8/IL8* contributes to tissue destruction and bone resorption, processes that are also regulated during orthodontic tooth movement (OTM) [51,52,53,54,55]. This underscores the importance of understanding how mechanical forces interact with oxidative stress in modulating these cytokines, especially in periodontal tissues, where both factors are at play during inflammation and tissue remodeling [54,56]. Also, contrary to these findings, in the case of *PTRGS2*/*COX2* gene expression and the related PGE2 secretion, tensile strain induced significantly higher gene expression in cells pre-exposed to higher dose of H_2_O_2_. Nevertheless, the current results indicate that the response towards tensile strains in cells pre-stimulated with H_2_O_2_ is considerably different to that of unstimulated cells.

### 3.3. Gene Expression Related to Bone Remodeling

Bone homeostasis involves bone formation and bone resorption, which are processes that maintain skeletal health. Osteoblasts are crucial for bone formation, and the expression of specific genes like *RUNX2*, *P2RX7* and *TNFRSF11B*/*OPG* plays a significant role in this process. The activation of *RUNX2* and *P2RX7* is known to enhance the expression of osteoblast markers and promote mineralization, while the *TNFRSF11B*/*OPG* protein acts as a decoy receptor for *RANKL*, inhibiting its osteoclast differentiation-inducing effect [57,58].

Oxidative stress is known to cause dysfunctional bone homeostasis, including osteoblast-induced osteogenesis and thus favoring bone resorption [59,60]. According to our results, OS generally had a negative effect on the expression of genes related to bone remodeling with a recovery tendency after 24 h post-incubation.

On the contrary, tensile strain is known to promote bone formation, which was also found herein. However, this stimulatory effect was not observable in groups of cells previously subjected to OS, suggesting and confirming a negative influence of OS on bone remodeling [22,61].

### 3.4. Apoptosis and Autophagy Related Gene Expression

Herein, we investigated the expressions of genes related to autophagy (*MAP1LC3A*/*LC3* and *BECN1*) and apoptosis (*CASP3* and *CASP8*). Growing evidence suggests that ROS can act as signaling molecules involved in cellular processes that regulate cell destiny, such as autophagy and apoptosis [60,62]. ROS activate autophagy, which helps cellular adaptation and reduces oxidative damage by breaking down and recycling damaged macromolecules and dysfunctional organelles [37,63]. In the same manner, it is also proven to influence apoptosis, maintaining tissue homeostasis by eliminating damaged cells [37]. Our results from the “direct”/“recovery” groups comparison support these findings by showing the upregulation of genes related to autophagy and apoptosis. Nevertheless, to draw more conclusions on how exactly OS triggers the regulation of signaling pathways that culminate in the regulation of autophagy and apoptosis, especially in relation to OTM, more studies are needed.

### 3.5. Study Limitations

To our knowledge, this is the first study examining the effects of oxidative stress on mechanically stimulated hOBs by combining established experimental setups for OS and mechanical tension application. However, it should be noted that OS and mechanical stimulation are much more complex, and this study is an in vitro simplification of more intricate processes. Our in vitro setup allowed us to break down complex in vivo situations by focusing on a single cell type (hOBs), one type of force (static tension), and one type of ROS, namely, H_2_O_2_. Nevertheless, this simplification did not consider confounding factors derived from the external environment, including but not limited to interactions with other cell types, the extracellular matrix, and the influence of various signaling molecules, including other reactive oxygen species/molecules and antioxidants [64,65]. Additionally, biological diversity should be considered, including cells from multiple donors of different sex and age groups. To address these complexities, more studies are needed. Nonetheless, this study serves as a valuable milestone for future research in this field.

### 3.6. Clinical Relevance

For periodontal patients, a distinct approach is imperative due to altered molecular responses. Therefore, studying and understanding molecular events in this population becomes crucial for comprehending therapeutic responses at the cellular and tissue levels. The results of this project can offer a good foundation for future clinical projects, especially in terms of novel methods in periodontal disease treatment combining orthodontic mechanical stimulation as a regenerative stimulus.

## 4. Materials and Methods

### 4.1. Primary Cell Culture

This study was conducted in accordance with the Declaration of Helsinki. Approval for the collection and use of human alveolar bone-derived osteoblasts (hOBs) was obtained from the ethics committee of the Ludwig-Maximilians-Universität München (project number 21-0931). Cells were obtained anonymously from a male donor undergoing orthognathic surgery exclusively for medical indications according to commonly accepted therapeutic standards. Written informed consent was obtained prior to cell sampling. The cells were isolated according to established procedures [66,67] and cultivated in low-glucose DMEM (21885025; Gibco/Life Technologies, Carlsbad, CA, USA) supplemented with 10% FBS (F7524; Sigma-Aldrich, St. Louis, MO, USA), 1 × MEM vitamins (M6895; Biochrom, Berlin, Germany) and 1% of antibiotic/antimycotic (15240-062; Life Technologies, Carlsbad, CA, USA). Cells were grown in a humidified atmosphere with 5% CO_2_ at 37 °C. Cell passaging was performed in regular intervals of 3 to 4 days using 0.05% trypsin-EDTA solution (59417C; Sigma-Aldrich, St. Louis, MO, USA). In all experiments cells from passages 5 or 6 have been seeded at a density of 2 × 10^5^ cells/well on 6-well collagen-I coated BioFlex^®^ culture plates (Flexcell Intl. Corp., Hillsborough, NC, USA).

### 4.2. Selection of H_2_O_2_ Concentration

To induce an oxidative stress-like environment, H_2_O_2_ (9681.4; Carl Roth GmbH + Co. KG, Karlsruhe, Germany) was added to the cell culture medium [17,18,28]. A dose–response experiment was carried out to determine the optimal H_2_O_2_ concentration, defined as the highest H_2_O_2_ concentration that can be applied in the experiments without affecting cell viability and proliferation [17,18,28]. For this purpose, concentrations ranging from 20 to 500 µM H_2_O_2_ were tested in hOB cell culture. Cells were seeded as described above and incubated over night to support equilibration. On the next day, the cell culture media was replaced by one containing different concentrations of H_2_O_2_ (20 µM, 50 µM, 100 µM, 200 µM, 500 µM) and cells were stimulated for an additional 24 h in the CO_2_ incubator. Wells with cells containing normal cell culture medium but otherwise treated identically served as controls.

The cytotoxic effect of H_2_O_2_ on cell viability was assessed as below. Additionally, cell viability was visually assessed using a live/dead viability/cytotoxicity assay as described below.

Cell cytotoxicity/viability assay. Cell culture supernatants were replaced with 2 mL/well of a resazurin-stock solution (alamarBlue™; Bio-Rad AbD Serotec GmbH, Puchheim, Germany) according to a previously published protocol [68]. After 2 h of incubation, cell culture supernatants and medium controls were collected and centrifuged, and the resazurin fluorescence of collected supernatants was determined using a fluorescence microplate reader (Varioscan; Thermo Electron Corporation, Vantaa, Finland) (560 nm excitation, 590 nm emission). For each measurement, “percentage reduction of resazurin” was calculated according to the manufacturer’s instructions [68]. Cell viability was calculated as normalized resazurin reduction relative to the control group.

Live/dead and apoptosis staining assays. The viability of hOBs incubated with the different H_2_O_2_ concentrations was assessed using a live/dead cell staining kit (L3224; ThermoFisher Scientific, Carlsbad, CA) according to the manufacturer’s instructions as previously published [20]. For apoptosis detection, one membrane of each experiment group was stained using the CellEvent™ Caspase-3/7 Green ReadyProbes^®^ reagent (R37111; Life Technologies, Carlsbad, CA, USA) according to the manufacturer’s instructions. After 30 min of incubation at room temperature in the dark, fluorescence microscopy was performed for all membranes using an EVOS*fl* fluorescence microscope (Invitrogen, Carlsbad, CA, USA) with 10× and 20× objectives.

### 4.3. Effect of Oxidative Stress Induction on Gene Expression in “Direct” and “Recovery” Setups

To investigate the effects of H_2_O_2_ treatment directly after 24 h of stimulation (“direct” group) and after additional 24 h post-incubation (“recovery” group), cells were seeded on collagen-I coated BioFlex^®^ plates in two identical setups in triplicates and incubated overnight as described above (Figure 4a). On the next day, cells were treated with cell culture medium containing 0 µM (i.e., control), 50 µM or 100 µM H_2_O_2_ and incubated in the CO_2_ incubator as described above. After 24 h, the cell lysates of the “direct” group were collected from each well using 750 µL RNA lysis buffer (R0160-1-50; Zymo, Irvine, CA, USA) according to the manufacturer’s instructions. Cell lysates were stored at −80 °C for RT-qPCR analysis. For the “recovery” group, fresh normal cell culture medium was added to all wells. After 24 h post-incubation, cell lysates were prepared as described and stored for RT-qPCR analysis.

### 4.4. Tensile Strain Application During the H_2_O_2_ “Recovery” Phase

To investigate the effects of H_2_O_2_ and tension force, for each experimental condition combination (control, 10%/15% tension, 50 µM/100 µM H_2_O_2_ + 10%/15% tension) two identical setups were used and processed in parallel: one set to assess cell proliferation and cell viability, the other one for gene expression measurement and ELISAs.

#### 4.4.1. Tensile Strain Application

After overnight incubation, cells were stimulated with or without 50/100 µM H_2_O_2_ and incubated for 24 h. Afterwards, the culture medium was removed, and wells were carefully washed twice with PBS. Fresh cell culture medium was then added as described previously [20]. The static tensile force application of 10% or 15% was conducted for an additional 24 h using a previously published in vitro tension model [20]. Controls were defined as wells without stretching and H_2_O_2_. For each experimental group, three biological replicates were allocated.

#### 4.4.2. Cell Proliferation and Cell Viability

After completion of the tensile force application, cell growth was assessed in triplicate using resazurin reduction in all experimental samples and their corresponding controls following the disassembly of tension setup as previously described with a shortened incubation time of 2 h [20,68]. For each measurement, the “percentage reduction of resazurin” was calculated.

In parallel with the tensile force application experiments, a resazurin standard curve was prepared as described previously to estimate cell proliferation during this experiment [68]. Briefly, hOBs from the 5th passage were diluted and seeded in triplicate (70,000/100,000/200,000/300,000/400,000/500,000 cells per well), in duplicate (800,000 cells per well) or in a single replication (900,000 cells per well). The cells were allowed to adhere overnight. The resazurin test was performed as described above [68]. For each measurement, the “percentage reduction of resazurin” was calculated. A standard curve (cell number vs. percentage reduction of resazurin) was established utilizing exponential regression (Microsoft Excel for Windows 365 MSO Version 2404, Microsoft Corporation, Redmond, WA, USA) (Figure 7), and the cell number was calculated for each well. The cell viability of hOBs from all experimental groups was then qualitatively assessed using a live/dead cell staining kit as described above.

#### 4.4.3. Sample Preparation

After completion of the experiment, the respective setup was disassembled, and cell culture supernatants were collected from the setup. Next, the adherent cells were washed twice with sterile PBS, and cell lysates were prepared as described in Section 4.3. Meanwhile, ELISA-specific aliquots were prepared from cell culture supernatants as previously published [20].

### 4.5. Gene Expression Analysis

The analysis of *PTGS2*/*COX2*, *IL6*, *CXCL8*/*IL8*, *RUNX2*, *CASP3*, *MAP1LC3A*/*LC3*, *BECN1*, *TNFRSF11B*/*OPG* and *P2RX7* gene expression following H_2_O_2_ stimulation and/or tensile strain application was carried out for all experimental groups according to previously described protocols [68]. In the following, a short summary of the sample preparation and quantitative RT-PCR process is given. A checklist based on the “Minimum Information for Publication of Quantitative Real-Time PCR Experiment” (MIQE) guidelines [69] is provided in Supplementary Materials S2.

Total RNA preparation and cDNA synthesis: RNA isolation and cDNA synthesis were performed as described previously [20,68] using the QuickRNA™ MicroPrep Kit (R1051; Zymo, Irvine, CA, USA) and SuperScript™ IV First-Strand Synthesis System (18091050, Thermo Fisher Scientific, Waltham, MA, USA), respectively.

PCR primer selection: Generally, primer sequences were selected from public sources for both genes of interest and potential reference genes (Supplementary Materials S2). All primer pairs used were tested in silico according to the MIQE guidelines [70] as previously published [68] (Supplementary Materials S2). Unmodified primers were synthesized by TIB Molbiol Syntheselabor GmbH (Berlin, Germany). Optimal annealing temperatures were determined with gradient PCR (TProfessional Gradient; Biometra, Göttingen, Germany) using the qPCR cycling program as specified in the MIQE checklist (Supplementary Materials S2). Primer specificity was confirmed by agarose gel electrophoresis. Primer efficiencies were evaluated using standard curves prepared from serial dilutions of cDNA, as specified in Supplementary Materials S2 and quantified in the LightCycler^®^ 480 (Roche Molecular Diagnostics, Basel, Switzerland) using the primer pairs detailed in Supplementary Materials S2.

**Figure 9:**
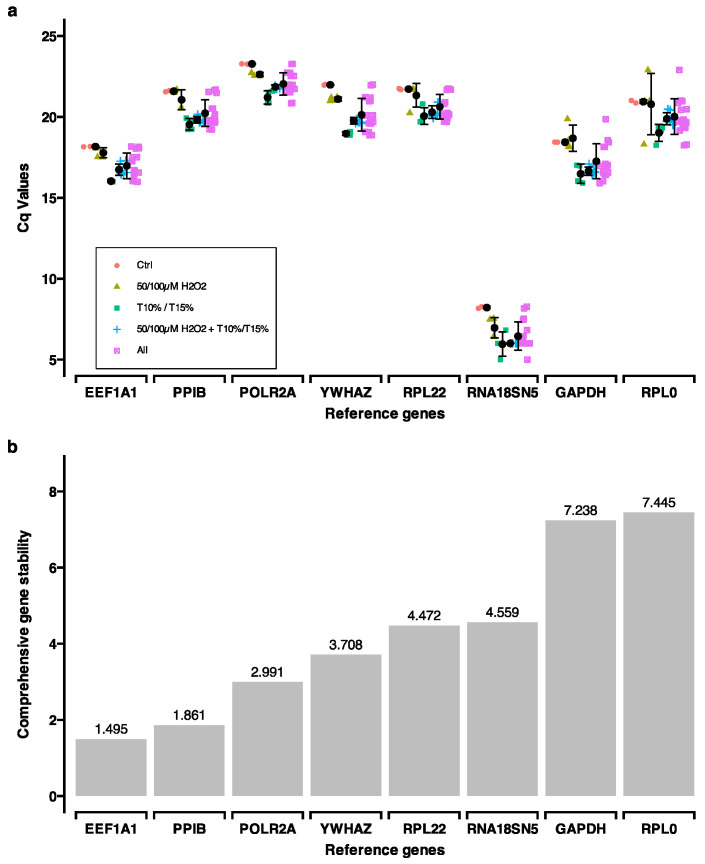
Reference gene selection was obtained with RefFinder. (**a**) Cq values for the panel of reference genes. Six quantitative real-time polymerase chain reaction (qPCR) runs were analyzed representing three biological replicates and two technical replicates each (Supplementary Materials S3). (**b**) Analysis of comprehensive gene stability for the panel of reference genes. Lower values indicate higher gene stability (Supplementary Materials S3).

Reference gene selection: A set of reference genes (*EEF1A1*, *GAPDH*, *POLR2A*, *PPIB*, *RNA18SN5*, *RPL0*, *RPL22* and *YWHAZ*) was selected from public sources [19,71]. The evaluation of these reference genes was carried out using cDNA sampled from control, tension application (15%), H_2_O_2_ stimulation for 24 h followed by 24 h recovery (50 µM and 100 µM H_2_O_2_), and H_2_O_2_ stimulation for 24 h followed by 24 h tension application (15%/50 µM H_2_O_2_, and 15%/100 µM H_2_O_2_). RT-qPCR was performed as described below using gene-specific primers (Supplementary Materials S2). The raw Cq values (Supplementary Materials S3) were analyzed using RefFinder [72,73]. This web-based tool integrates four different algorithms (BestKeeper [74], NormFinder [75], geNorm [76], and comparative ΔCt method [77]) to compare and rank candidate reference genes. Based on the rankings and the two most stable genes (*PPIB*, *EEF1A1*) were used as reference genes in RT-qPCR (Figure 9).

Quantitative PCR was carried out using the LightCycler^®^ 480 SYBR Green I Master kit (04887352001; Roche Diagnostics GmbH, Mannheim, Germany) as per the manufacturer’s protocol, with 5 µL of cDNA (1:10 prediluted) in each PCR reaction. Further details regarding the RT-qPCR reaction conditions are outlined in the MIQE checklist (Supplementary Materials S2). The PCR primer specification is summarized in Table 3.

Gene expression calculation: The expression level of target genes was quantified applying the 2^−ΔΔCq^ method [76,84] using the average (geometric mean) of the selected reference genes (*PPIB* and *EEF1A1*). For each tension/H_2_O_2_ concentration combination, six RT-qPCR reactions were analyzed, representing three biological replicates with two technical replicates each (*n* = 3, *n* = 6).

### 4.6. Enzyme-Linked Immunosorbent Assay

Cell culture supernatants from all wells were collected for ELISA as described above. IL6 and PGE2 concentrations were determined using specific ELISA systems; for IL6, the DuoSet human IL6 ELISA kit (DY206-05; R&D Systems, Minneapolis, MN, USA) was used, whereas PGE2 was determined using the “PGE2 High Sensitivity ELISA kit” (ADI-931-001; Enzo Life Sciences AG, Lausen, CH). All measurements were conducted using a microplate reader (Varioscan, Thermo Electron Corporation, Vantaa, Finland). For each marker molecule/experimental condition combination, three biological replicates were measured twice. The measurements were reported as “pg per 100,000 cells” using the well-specific cell numbers determined above.

### 4.7. Statistics

Descriptive statistics of the gene expression and ELISA results are reported as mean and standard deviation (SD), median and minimum/maximum. All calculations were based on three biological replicates with two technical replicates for each gene/experimental condition combination. For each gene locus and marker molecule, differences between the different tensile strain magnitudes and durations were evaluated using the Kruskal–Wallis test followed by multiple comparisons with Bonferroni correction applied (*p*_adj_.). All statistical procedures were carried out using IBM SPSS Statistics 29 (IBM Corp., Armonk, NY, USA) and were two-tailed considering *p*_adj_ values < 0.05 as significant.

## 5. Conclusions

Our results suggest that OS might have a significant impact on OTM through the regulation of bone remodeling-, inflammation-, autophagy-, and apoptosis-related genes. Additionally, this study highlights the necessity of considering the complexity of OS and mechanical stimulation in a more comprehensive manner and environment, accounting for interactions with various cell types, extracellular matrix components, and a range of other signaling molecules, normally present in in vivo situation. Despite its in vitro limitations and the simplified nature of the model, this work provides a valuable milestone for improving our understanding of these complex processes and guiding further clinical studies in the field. Understanding the interplay between OS and mechanical stimulation in these patients is essential for improving clinical outcomes and minimizing risks such as delayed healing, compromised bone remodeling, and exacerbated inflammatory responses.

## Figures and Tables

**Table 1:** Summary statistics and comparisons of the effects of 50 µM and 100 µM H_2_O_2_ on gene expression (*CXCL8/IL8, IL6, PTGS2/COX2, CASP3, CASP8, MAP1LC3A/LC3, BECN1, TNFRSF11B/OPG*, and *P2RX7*) reported as fold change in primary hOBs immediately after H_2_O_2_ incubation (“direct”) and 24 h post-incubation (“recovery”). *p*-values were obtained with the Kruskal–Wallis test (KW) and adjusted by Bonferroni correction for multiple tests (adjusted *p*, *p*_adj_.).

Analyte	Treatment	Mean	SD	Median	Min	Max	K-W of Treatment		
							*p* Value	Post-Hoc Test vs. Ctrl (*p*_adj_.)	Sig. ^a^
Direct									
*CXCL8/IL8* (FC)	Ctrl	1.00	0.06	1.00	0.94	1.12	0.004		**
	50 µM	0.97	0.17	0.96	0.81	1.26		1.000	n.s.
	100 µM	1.41	0.16	1.38	1.26	1.69		0.018	*
*IL6* (FC)	Ctrl	1.02	0.24	1.00	0.75	1.37	0.001		**
	50 µM	1.49	0.36	1.45	1.01	1.96		0.249	n.s.
	100 µM	2.47	0.47	2.65	1.54	2.86		0.001	**
*PTGS2/COX2* (FC)	Ctrl	1.01	0.13	1.00	0.86	1.17	<0.001		***
	50 µM	2.15	0.06	2.15	2.08	2.22		0.153	n.s.
	100 µM	3.39	0.43	3.23	2.99	4.08		<0.001	***
*RUNX2* (FC)	Ctrl	1.00	0.06	1.00	0.94	1.07	0.003		**
	50 µM	0.64	0.14	0.60	0.50	0.83		0.015	*
	100 µM	0.61	0.21	0.71	0.31	0.79		0.007	**
*CASP3* (FC)	Ctrl	1.02	0.21	1.00	0.76	1.28	0.205		n.s.
	50 µM	0.74	0.29	0.64	0.49	1.11		0.350	n.s.
	100 µM	0.97	0.31	1.11	0.38	1.19		1.000	n.s.
*CASP8* (FC)	Ctrl	1.01	0.19	1.00	0.80	1.31	0.026		*
	50 µM	1.35	0.39	1.55	0.81	1.65		0.248	n.s.
	100 µM	1.57	0.14	1.60	1.39	1.77		0.024	*
*MAP1LC3A/LC3* (FC)	Ctrl	1.00	0.10	1.00	0.90	1.17	0.002		**
	50 µM	1.20	0.05	1.20	1.13	1.28		0.144	n.s.
	100 µM	1.28	0.05	1.28	1.21	1.34		0.001	**
*BECN1* (FC)	Ctrl	1.00	0.10	1.00	0.90	1.19	0.001		**
	50 µM	1.17	0.05	1.17	1.08	1.24		0.388	n.s.
	100 µM	1.64	0.23	1.52	1.44	1.96		0.001	**
*TNFRSF11B/OPG* (FC)	Ctrl	1.01	0.13	0.98	0.86	1.25	0.128		n.s.
	50 µM	0.94	0.25	1.05	0.63	1.20		1.000	n.s.
	100 µM	0.84	0.11	0.86	0.72	0.95		0.154	n.s.
*P2RX7* (FC)	Ctrl	1.01	0.16	1.00	0.83	1.28	0.003		**
	50 µM	0.56	0.16	0.60	0.32	0.73		0.024	*
	100 µM	0.49	0.07	0.46	0.42	0.58		0.004	**
Recovery									
*CXCL8/IL8* (FC)	Ctrl	1.00	0.03	1.00	0.96	1.05	<0.001		***
	50 µM	0.83	0.06	0.83	0.73	0.94		0.154	n.s.
	100 µM	1.68	0.39	1.51	1.33	2.21		0.154	n.s.
*IL6* (FC)	Ctrl	1.00	0.05	1.00	0.92	1.07	0.142		n.s.
	50 µM	0.98	0.09	0.97	0.84	1.09		1.000	n.s.
	100 µM	1.07	0.09	1.07	0.98	1.21		0.665	n.s.
*PTGS2/COX2* (FC)	Ctrl	1.00	0.04	1.00	0.93	1.07	<0.001		***
	50 µM	0.69	0.09	0.70	0.57	0.78		0.154	n.s.
	100 µM	2.10	0.32	2.08	1.73	2.48		0.154	n.s.
*RUNX2* (FC)	Ctrl	1.00	0.09	1.01	0.84	1.08	0.024		*
	50 µM	0.84	0.09	0.83	0.78	1.02		0.028	*
	100 µM	0.86	0.12	0.89	0.64	0.95		0.126	n.s.
*CASP3* (FC)	Ctrl	1.00	0.01	1.00	0.99	1.01	0.001		**
	50 µM	0.89	0.12	0.88	0.75	1.06		0.575	n.s.
	100 µM	2.83	0.68	2.76	2.00	3.66		0.067	n.s.
*CASP8* (FC)	Ctrl	1.00	0.01	1.00	0.98	1.01	0.005		**
	50 µM	0.91	0.18	0.89	0.69	1.17		1.000	n.s.
	100 µM	1.24	0.15	1.24	1.09	1.51		0.038	*
*MAP1LC3A/LC3 (FC)*	Ctrl	1.01	0.12	1.00	0.88	1.19	0.001		**
	50 µM	0.61	0.01	0.61	0.60	0.63		0.091	n.s.
	100 µM	1.17	0.05	1.17	1.11	1.24		0.388	n.s.
*BECN1* (FC)	Ctrl	1.00	0.02	1.00	0.96	1.02	0.003		**
	50 µM	0.87	0.19	0.86	0.66	1.12		1.000	n.s.
	100 µM	2.71	0.42	2.68	2.21	3.19		0.028	*
*TNFRSF11B/OPG* (FC)	Ctrl	1.00	0.02	1.00	0.97	1.03	<0.001		***
	50 µM	0.70	0.14	0.69	0.52	0.88		0.154	n.s.
	100 µM	1.75	0.04	1.75	1.70	1.80		0.154	n.s.
*P2RX7* (FC)	Ctrl	1.00	0.03	1.00	0.96	1.03	<0.001		***
	50 µM	0.86	0.05	0.87	0.78	0.92		0.154	n.s.
	100 µM	0.57	0.03	0.57	0.52	0.62		0.154	n.s.

^a^ Sig., significance; *, *p*_adj_. < 0.05; **, *p*_adj_. < 0.01; ***, *p*_adj_. < 0.001; n.s., not significant.

**Table 2:** Summary statistics and comparison of the expression of genes and metabolites in mechanically stimulated cells with and without previous H_2_O_2_ stimulation. Data from RT-qPCR experiments are given as fold change, and data from ELISA (PGE2 and IL6) as “pg/100,000 cells”. *p*-values were obtained with the Kruskal–Wallis test (K-W) and adjusted by Bonferroni correction for multiple tests (adjusted *p*, *p*_adj_.).

Analyte	Treatment	Mean	SD	Median	Min	Max	K-W of Treatment		
							*p* Value	Post-Hoc Test vs. Ctrl (*p*_adj_.)	Sig.^a^
*CXCL8/IL8* (FC)	Ctrl	1.00	0.03	1.00	0.96	1.05	<0.001		***
	T15%	2.74	0.34	2.85	2.08	3.00		0.001	**
	50 µM/T15%	2.37	0.90	2.36	1.19	3.38		0.010	*
	100 µM/T15%	1.55	0.30	1.49	1.25	1.91		0.397	n.s.
*IL6* (FC)	Ctrl	1.00	0.05	1.00	0.92	1.07	<0.001		***
	T15%	2.01	0.42	1.93	1.55	2.55		0.022	*
	50 µM/T15%	1.17	0.08	1.17	1.06	1.30		0.989	n.s.
	100 µM/T15%	0.45	0.06	0.42	0.40	0.58		0.784	n.s.
*PTGS2/COX2* (FC)	Ctrl	1.00	0.04	1.00	0.93	1.07	<0.001		***
	T15%	2.03	0.34	2.13	1.56	2.35		0.024	*
	50 µM/T15%	1.49	0.08	1.48	1.38	1.59		0.754	n.s.
	100 µM/T15%	3.06	0.48	2.83	2.66	3.69		<0.001	***
*RUNX2* (FC)	Ctrl	1.00	0.09	1.01	0.84	1.08	<0.001		***
	T15%	1.09	0.12	1.07	0.95	1.25		1.000	n.s.
	50 µM/T15%	0.52	0.10	0.50	0.42	0.71		0.042	*
	100 µM/T15%	0.49	0.07	0.51	0.40	0.56		0.042	*
*CASP3* (FC)	Ctrl	1.00	0.01	1.00	0.99	1.01	<0.001		***
	T15%	2.86	0.09	2.89	2.69	2.93		<0.001	***
	50 µM/T15%	1.63	0.34	1.69	1.21	2.06		0.326	n.s.
	100 µM/T15%	1.89	0.49	1.96	1.24	2.57		0.075	n.s.
*CASP8* (FC)	Ctrl	1.00	0.01	1.00	0.98	1.01	0.008		**
	T15%	1.64	0.33	1.68	1.19	2.00		0.009	**
	50 µM/T15%	1.25	0.36	1.32	0.74	1.78		1.000	n.s.
	100 µM/T15%	1.45	0.17	1.42	1.27	1.72		0.086	n.s.
*MAP1LC3A/LC3 (FC)*	Ctrl	1.01	0.12	1.00	0.88	1.19	<0.001		***
	T15%	2.14	0.27	2.18	1.80	2.43		<0.001	***
	50 µM/T15%	1.77	0.43	1.63	1.37	2.45		0.033	*
	100 µM/T15%	1.47	0.22	1.45	1.14	1.80		0.345	n.s.
*BECN1* (FC)	Ctrl	1.00	0.02	1.00	0.96	1.02	<0.001		***
	T15%	2.98	0.14	2.97	2.80	3.15		<0.001	***
	50 µM/T15%	2.65	0.21	2.64	2.34	2.98		0.037	*
	100 µM/T15%	2.44	0.24	2.44	1.99	2.69		0.396	n.s.
*TNFRSF11B/OPG* (FC)	Ctrl	1.00	0.02	1.00	0.97	1.03	<0.001		***
	T15%	1.81	0.28	1.77	1.49	2.25		<0.001	***
	50 µM/T15%	1.25	0.12	1.24	1.06	1.39		0.037	*
	100 µM/T15%	0.84	0.16	0.83	0.61	1.11		0.396	n.s.
*P2RX7* (FC)	Ctrl	1.00	0.03	1.00	0.96	1.03	<0.001		***
	T15%	1.45	0.12	1.44	1.31	1.66		0.119	n.s.
	50 µM/T15%	1.11	0.20	1.05	0.90	1.35		1.000	n.s.
	100 µM/T15%	0.40	0.06	0.37	0.34	0.51		0.272	n.s.
PGE2 (pg/100,000 cells)	Ctrl	91.91	6.85	93.31	82.88	100.73	<0.001		***
	T15%	139.88	4.20	140.10	135.10	145.15		0.850	n.s.
	50 µM/T15%	169.36	10.80	166.20	157.58	186.22		0.012	*
	100 µM/T15%	185.15	7.16	184.72	177.37	193.31		<0.001	***
IL6 (pg/100,000 cells)	Ctrl	230.68	16.07	236.51	205.93	245.50	<0.001		***
	T15%	281.39	27.81	281.23	253.51	313.45		0.086	n.s.
	50 µM/T15%	301.51	19.78	304.30	270.36	329.25		0.009	**
	100 µM/T15%	226.57	13.24	225.64	207.95	248.14		1.000	n.s.

^a^ Sig., significance; *, *p*_adj_. < 0.05; **, *p*_adj_. < 0.01; ***, *p*_adj_. < 0.001; n.s., not significant.

**Table 3:** Specification of the PCR primers used for gene quantification.

Gene	GenBank Accession Number	Primer Sequence (f: 5′-Forward Primer-3′; r: 5′-Reverse Primer-3′)	Annealing Temp. (°C)	Amplicon Size (bp)	Reference
*PTGS2/COX2*	NM_000963.4	f: AAGCCTTCTCTAACCTCTCC r: GCCCTCGCTTATGATCTGTC	58	234	[68,78]
*IL6*	NM_000600.5	f: TGGCAGAAAACAACCTGAACC r: TGGCTTGTTCCTCACTACTCTC	58	168	[68,78]
*CXCL8/IL8*	NM_000584.4	f: CAGAGACAGCAGAGCACACAA r: TTAGCACTCCTTGGCAAAAC	55	170	[79]
*RUNX2*	NM_001015051.4	f: GCGCATTCCTCATCCCAGTA r: GGCTCAGGTAGGAGGGGTAA	58	176	[67,68]
*BECN1*	NM_003766.5	f: AGGTTGAGAAAGGCGAGACA r: AATTGTGAGGACACCCAAGC	58	196	[80]
*MAP1LC3A/LC3*	NM_032514.4	f: CGTCCTGGACAAGACCAAGT r: TCCTCGTCTTTCTCCTGCTC	58	183	[80]
*CASP3*	NM_004346.4	f: TGGAGGCCGACTTCTTGTAT r: ACTGTTTCAGCATGGCACAA	58	111	[81]
*CASP8*	NM_001228.5	f: GGAGGAGTTGTGTGGGGTAA r: CCTGCATCCAAGTGTGTTCC	58	207	[82]
*TNFRSF11B/OPG*	NM_002546.4	f: TCAAGCAGGAGTGCAATCG r: AGAATGCCTCCTCACACAGG	64	342	[83]
*P2RX7*	NM_002562.6	f: AGTGCGAGTCCATTGTGGAG r: CATCGCAGGTCTTGGGACTT	58	143	[67]
*EEF1A1*	NM_001402.6	f: CCTGCCTCTCCAGGATGTCTAC r: GGAGCAAAGGTGACCACCATAC	61	105	[19,20]
*PPIB*	NM_000942.5	f: TTCCATCGTGTAATCAAGGACTTC r: GCTCACCGTAGATGCTCTTTC	55	88	[19,20]

## Data Availability

All authors confirm that all related data supporting the findings of this study are given in the article and its Supplementary Materials.

## Supplementary Materials

The following supporting information can be downloaded at: https://www.mdpi.com/article/10.3390/ijms252413525/s1, Supplement 1: Influence of tension during recovery (Table S1 and Graphics) and Live/Dead Cell Staining (Images), Supplement 2: MIQE Reporting (Table S2.1: MIQE Checklist for the RT-qPCR Workflow, Table S2.2: In Silico Analysis of the RT-qPCR Primer, Table S2.3: Primer Validation by RT-qPCR), References [19,20,66,67,68,71,78,79,80,81,82,83] are cited here, Supplement 3: RefFinder Results (1: Raw data, Cq Values, 2: RefFinder Summary, 2.1.: Delta CT, 2.2.: BestKeeper, 2.3.: normFinder, 2.4.: Genorm), References [72,73,74,75,76,77] are cited here.

## Abbreviations

BECN1	Beclin 1
CASP3	Caspase 3
CASP8	Caspase 8
ELISA	Enzyme-linked immunosorbent assay
FC	Fold change
H_2_O_2_	Hydrogen peroxide
hOBs	Human osteoblasts
IL6	Interleukin 6
CXCL8/IL8	C-X-C Motif Chemokine Ligand 8 (aka: IL8, interleukin-8)
MAP1LC3A/LC3	Microtubule Associated Protein 1 Light Chain 3 Alpha (aka: LC3)
MIQE	Minimum Information for Publication of Quantitative Real-Time PCR Experiment
TNFRSF11B/OPG	TNF Receptor Superfamily Member 11b (aka: OPG, osteoprotegerin)
OS	Oxidative stress
OTM	Orthodontic tooth movement
P2RX7	Purinergic Receptor P2X 7
*p*_adj_.	Adjusted *p*-value
PGE2	Prostaglandin E2
PTGS2/COX2	Prostaglandin-Endoperoxide Synthase 2 (aka: COX2, cyclooxygenase 2)
ROS	Reactive oxygen species
RT-qPCR	Reverse-transcriptase quantitative polymerase chain reaction
RUNX2	RUNX Family Transcription Factor 2
