# Supplementary material for: Investigation of Oxidative-Stress Impact on Human Osteoblasts During Orthodontic Tooth Movement Using an In Vitro Tension Model
Source: Int J Mol Sci. 2024 Dec 17;25(24):13525. doi: 10.3390/ijms252413525 (PMC11677893; doi:10.3390/ijms252413525)

Supplement 3 to manuscript

"Investigation of Oxidative-Stress Impact on Orthodontic Tooth Movement Using an In Vitro Tension Model"

URL: https://www.ciidirsinaloa.com.mx/RefFinder-master/ (2024-04-26)

RefFinder is a user-friendly web-based comprehensive tool developed for evaluating and screening reference genes from extensive experimental datasets. It integrates the currently available major computational programs (geNorm, Normfinder, BestKeeper, and the comparative ΔCt method) to compare and rank the tested candidate reference genes. Based on the rankings from each program, it assigns an appropriate weight to an individual gene and calculated the geometric mean of their weights for the overall final ranking.

Citation: F Xie, P Xiao, D Chen, L Xu, B Zhang. 2012. miRDeepFinder: a miRNA analysis tool for deep sequencing of plant small RNAs. Plant Mol Biol 80 (1), 75-84.

References

1. BestKeeper: Pfaffl MW, Tichopad A, Prgomet C, Neuvians TP. 2004. Determination of stable housekeeping genes, differentially regulated target genes and sample integrity: BestKeeper--Excel-based tool using pair-wise correlations. Biotechnology Lett 26:509-515.
2. NormFinder: Andersen CL, Jensen JL, Orntoft TF. 2004. Normalization of real-time quantitative reverse transcription-PCR data: a model-based variance estimation approach to identify genes suited for normalization, applied to bladder and colon cancer data sets. Cancer Res 64:5245-5250.
3. Genorm: Vandesompele J, De Preter K, Pattyn F, Poppe B, Van Roy N, De Paepe A, Speleman F. 2002. Accurate normalization of real-time quantitative RT-PCR data by geometric averaging of multiple internal control genes. Genome Biol 3:RESEARCH0034.
4. The comparative delta-Ct method: Silver N, Best S, Jiang J, Thein SL. 2006. Selection of housekeeping genes for gene expression studies in human reticulocytes using real-time PCR. BMC Mol Biol 7:33.

Contents

1 Raw data (Cq values) 2

2 RefFinder – Summary 3

2.1 Delta CT 5

2.2 BestKeeper 6

2.3 normFinder 7

2.4 Genorm 8

1 Raw data (Cq values)

Condition	Group	GAPDH	RPL22	RPL0	RNA18SN5	PPIB	YWHAZ	EEF1A1	POLR2A
Control	0	18.43	21.75	21.01	8.17	21.62	22.00	18.17	23.28
Control	0	18.44	21.68	20.86	8.27	21.55	21.96	18.15	23.26
H_2O_2 100µM	1	18.56	21.69	22.89	6.40	20.51	21.00	17.50	22.53
H_2O_2 100µM	1	19.86	20.24	18.30	6.44	20.52	20.97	17.52	22.52
H_2O_2 50µM	1	18.14	21.67	20.97	7.53	21.49	21.21	18.04	22.71
H_2O_2 50µM	1	18.14	21.71	20.97	7.47	21.69	21.19	18.06	22.73
H_2O_2 100µM + T10%	3	16.97	20.91	20.42	6.00	20.15	20.09	17.26	21.73
H_2O_2 100µM + T10%	3	17.09	20.91	20.48	6.00	20.17	20.10	17.29	21.74
H_2O_2 50µM + T10%	3	16.42	20.05	19.65	6.00	19.80	19.59	16.42	21.73
H_2O_2 50µM + T10%	3	16.43	19.91	19.49	6.00	19.69	19.60	16.44	21.73
T10%	2	16.97	20.80	19.33	6.82	19.23	18.86	15.99	20.83
T10%	2	17.01	19.98	18.24	6.00	19.22	18.87	16.04	20.87
H_2O_2 100µM + T15%	3	16.61	20.21	19.83	6.00	19.65	19.67	16.69	21.95
H_2O_2 100µM + T15%	3	16.55	20.14	19.77	6.00	19.66	19.68	16.71	21.97
H_2O_2 50µM + T15%	3	16.56	20.10	19.69	6.00	19.72	19.67	16.53	21.96
H_2O_2 50µM + T15%	3	16.50	20.09	19.72	6.00	19.73	19.66	16.56	21.99
T15%	2	16.03	19.71	19.31	5.00	19.92	19.05	16.02	21.52
T15%	2	15.91	19.70	19.15	6.00	19.75	19.08	16.05	21.58

	Ctrl (N= 2)	H_2O_2 100µM, H_2O_2 50µM (N= 4)	T10%, T15% (N= 4)	H_2O_2 50/100µM + T10%, H_2O_2 50/100µM + T15% (N= 8)	All (N= 18)
Group	0	1	2	3	5
GAPDH					
Mean (SD)	18 (0.0071)	19 (0.81)	16 (0.59)	17 (0.25)	17 (1.1)
Median [Min, Max]	18 [18, 18]	18 [18, 20]	17 [16, 17]	17 [16, 17]	17 [16, 20]
RPL22					
Mean (SD)	22 (0.049)	21 (0.73)	20 (0.52)	20 (0.39)	21 (0.77)
Median [Min, Max]	22 [22, 22]	22 [20, 22]	20 [20, 21]	20 [20, 21]	20 [20, 22]
RPL0					
Mean (SD)	21 (0.11)	21 (1.9)	19 (0.52)	20 (0.37)	20 (1.1)
Median [Min, Max]	21 [21, 21]	21 [18, 23]	19 [18, 19]	20 [19, 20]	20 [18, 23]
RNA18SN5					
Mean (SD)	8.2 (0.071)	7.0 (0.62)	6.0 (0.74)	6.0 (0)	6.5 (0.87)
Median [Min, Max]	8.2 [8.2, 8.3]	7.0 [6.4, 7.5]	6.0 [5.0, 6.8]	6.0 [6.0, 6.0]	6.0 [5.0, 8.3]
PPIB					
Mean (SD)	22 (0.049)	21 (0.63)	20 (0.36)	20 (0.21)	20 (0.82)
Median [Min, Max]	22 [22, 22]	21 [21, 22]	19 [19, 20]	20 [20, 20]	20 [19, 22]
YWHAZ					
Mean (SD)	22 (0.028)	21 (0.13)	19 (0.12)	20 (0.21)	20 (1.0)
Median [Min, Max]	22 [22, 22]	21 [21, 21]	19 [19, 19]	20 [20, 20]	20 [19, 22]
EEF1A1					
Mean (SD)	18 (0.014)	18 (0.31)	16 (0.026)	17 (0.35)	17 (0.79)
Median [Min, Max]	18 [18, 18]	18 [18, 18]	16 [16, 16]	17 [16, 17]	17 [16, 18]
POLR2A					
Mean (SD)	23 (0.014)	23 (0.11)	21 (0.41)	22 (0.13)	22 (0.69)
Median [Min, Max]	23 [23, 23]	23 [23, 23]	21 [21, 22]	22 [22, 22]	22 [21, 23]

2 RefFinder – Summary

Ranking Order (Better--Good--Average)

Method	1	2	3	4	5	6	7	8
Delta CT	EEF1A1	PPIB	YWHAZ	POLR2A	RPL22	RNA18SN5	GAPDH	RPL0
BestKeeper	POLR2A	RNA18SN5	PPIB	RPL22	EEF1A1	RPL0	YWHAZ	GAPDH
Normfinder	EEF1A1	PPIB	YWHAZ	RPL22	POLR2A	RNA18SN5	GAPDH	RPL0
Genorm	PPIB | EEF1A1		YWHAZ	POLR2A	RPL22	RNA18SN5	GAPDH	RPL0
**Recommended comprehensive ranking**	**EEF1A1**	**PPIB**	**POLR2A**	**YWHAZ**	**RPL22**	**RNA18SN5**	**GAPDH**	**RPL0**

Comprehensive Ranking:

Genes	Geomean of ranking values
EEF1A1	1.50
PPIB	1.86
POLR2A	2.99
YWHAZ	3.71
RPL22	4.47
RNA18SN5	4.56
GAPDH	7.24
RPL0	7.44

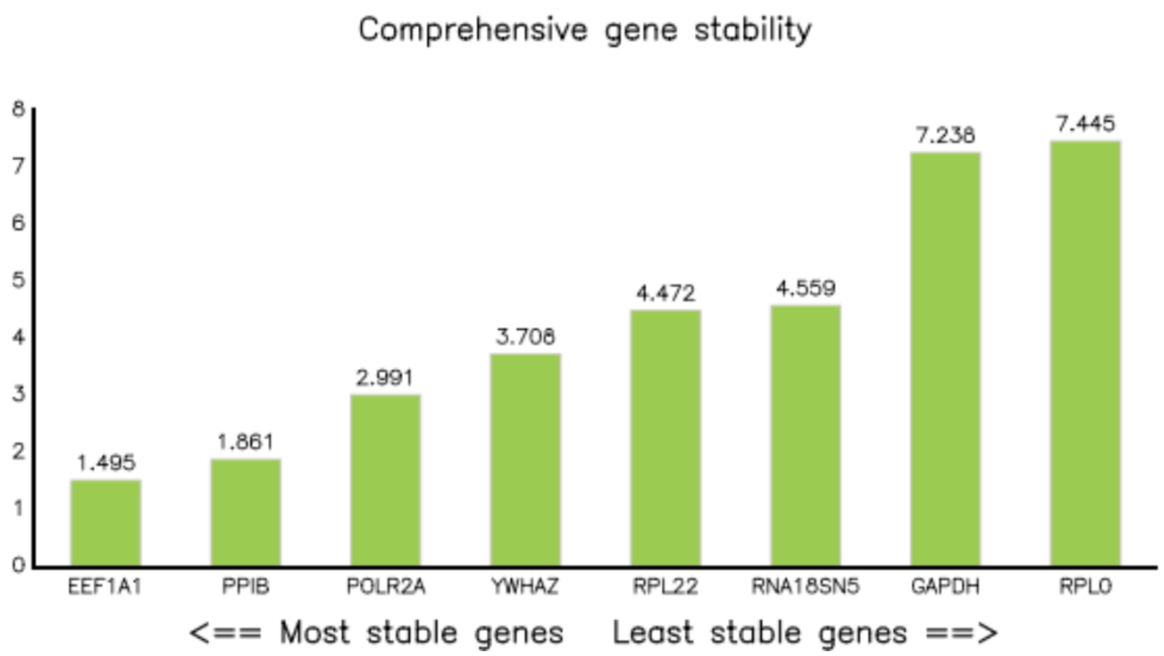

Supplement 3: RefFinder results

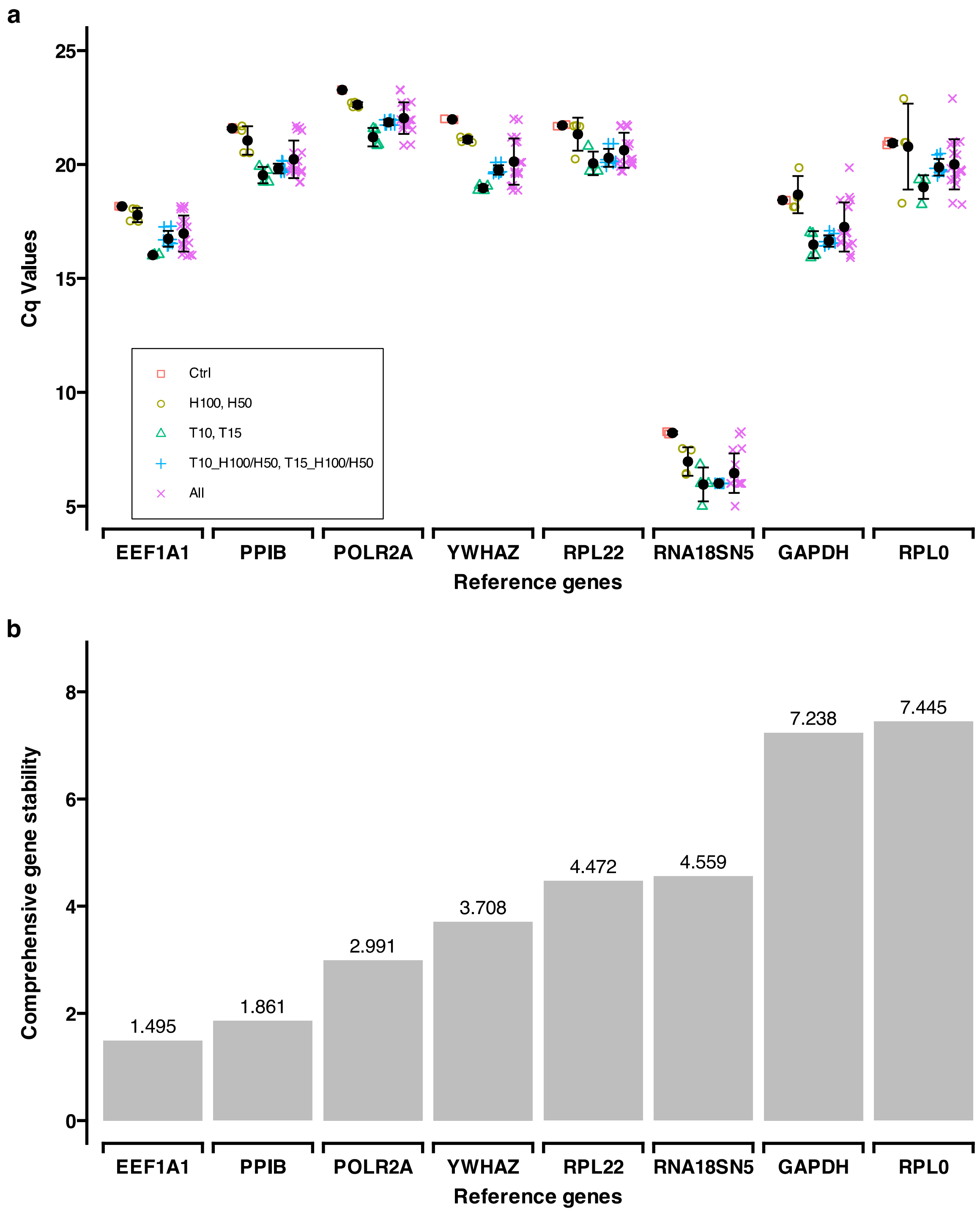

Summary figure. Reference gene stability - comparison between qPCR raw data (see section 1) RefFinder results. (a) Cq values for the panel of reference genes, using samples exposed to different experimental conditions (Ctrl, control; H100/H50, 50 µM or 100 µM H_2O_2 incubation for 24 h and 24 h recovery; T10 / T15, 10 % or 15 % static stretching applied for 24 h; T10_H100/H50 resp. T15_H100/H50, 50 µM or 100 µM H_2O_2 incubation for 24 h followed by 24 h static stretching at 10 % or 15 %; All, average expression for all four experimental regimes). Two qPCR runs of each combination were analyzed representing one biological sample with two technical replicates each. (b) Comprehensive gene stability analysis for the panel of reference genes. Lower values indicate higher gene stability.

Supplement 3: RefFinder results

2.1 Delta CT

Genes	Average of STDEV
EEF1A1	0.48
PPIB	0.53
YWHAZ	0.54
POLR2A	0.56
RPL22	0.57
RNA18SN5	0.66
GAPDH	0.81
RPL0	0.92

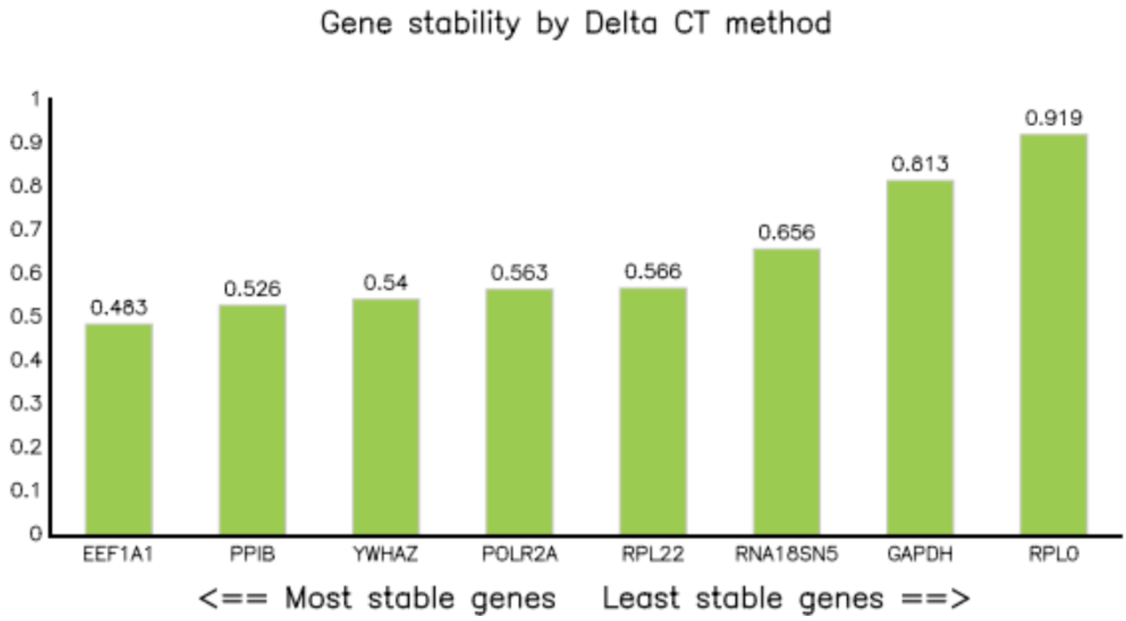

2.2 BestKeeper

CP data of housekeeping Genes by BEST KEEPER

	GAPDH	RPL22	RPL0	RNA18SN5	PPIB	YWHAZ	EEF1A1	POLR2A
n	18	18	18	18	18	18	18	18
geo Mean [CP]	17.23	20.61	19.98	6.40	20.21	20.10	16.95	22.02
AR Mean [CP]	17.26	20.63	20.00	6.45	20.23	20.13	16.97	22.04
min [CP]	15.91	19.70	18.24	5.00	19.22	18.86	15.99	20.83
max [CP]	19.86	21.75	22.89	8.27	21.69	22.00	18.17	23.28
std dev [+/- CP]	0.89	0.68	0.84	0.67	0.67	0.84	0.69	0.54
CV [% CP]	5.17	3.30	4.20	10.35	3.31	4.18	4.09	2.43
min [x-fold]	-2.49	-1.88	-3.33	-2.64	-1.99	-2.36	-1.95	-2.29
max [x-fold]	6.21	2.20	7.54	3.66	2.79	3.73	2.33	2.39
std dev [+/- x-fold]	1.86	1.60	1.79	1.59	1.59	1.79	1.62	1.45

Pearson correlation coefficient (r) by BEST KEEPER

	GAPDH	RPL22	RPL0	RNA18SN5	PPIB	YWHAZ	EEF1A1	POLR2A
RPL22	0.684	-	-	-	-	-	-	-
p-value	0.002	-	-	-	-	-	-	-
RPL0	0.351	0.805	-	-	-	-	-	-
p-value	0.153	0.001	-	-	-	-	-	-
RNA18SN5	0.664	0.826	0.466	-	-	-	-	-
p-value	0.003	0.001	0.051	-	-	-	-	-
PPIB	0.704	0.825	0.612	0.811	-	-	-	-
p-value	0.001	0.001	0.007	0.001	-	-	-	-
YWHAZ	0.817	0.833	0.642	0.818	0.936	-	-	-
p-value	0.001	0.001	0.004	0.001	0.001	-	-	-
EEF1A1	0.794	0.868	0.658	0.786	0.935	0.973	-	-
p-value	0.001	0.001	0.003	0.001	0.001	0.001	-	-
POLR2A	0.689	0.695	0.609	0.734	0.901	0.954	0.900	-
p-value	0.002	0.001	0.007	0.001	0.001	0.001	0.001	-

Pearson correlation coefficient (r)

BestKeeper vs.	GAPDH	RPL22	RPL0	RNA18SN5	PPIB	YWHAZ	EEF1A1	POLR2A
coeff. of corr. [r]	0.803	0.926	0.689	0.907	0.933	0.968	0.956	0.889
p-value	0.001	0.001	0.002	0.001	0.001	0.001	0.001	0.001

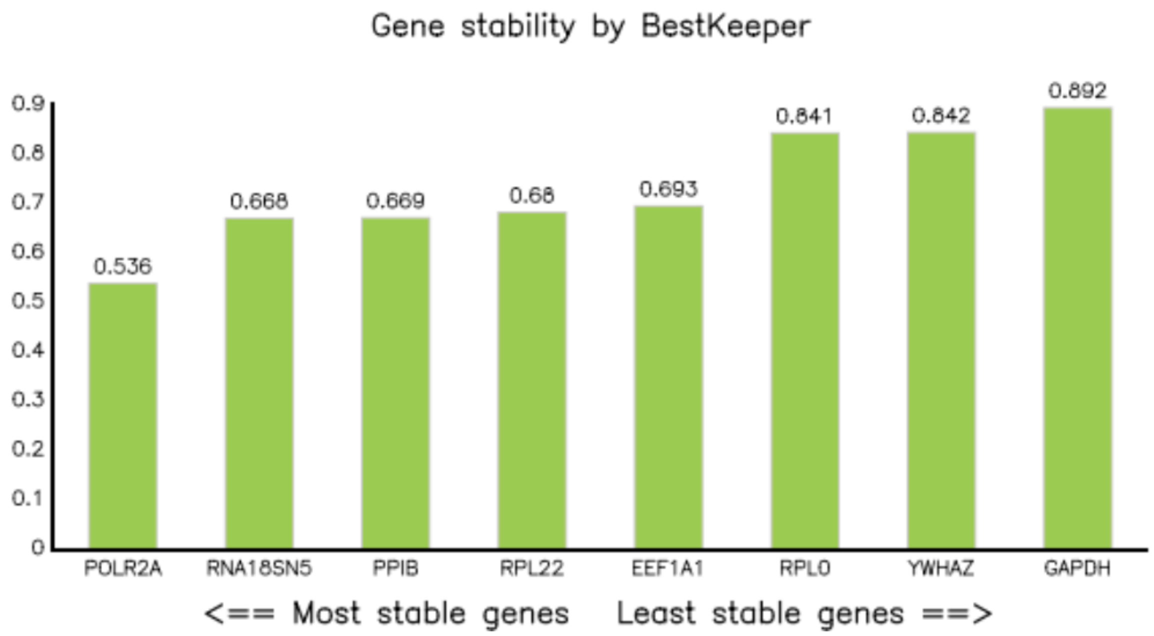

2.3 normFinder

Gene name	Stability value
EEF1A1	0.106
PPIB	0.262
YWHAZ	0.278
RPL22	0.295
POLR2A	0.331
RNA18SN5	0.488
GAPDH	0.714
RPL0	0.846

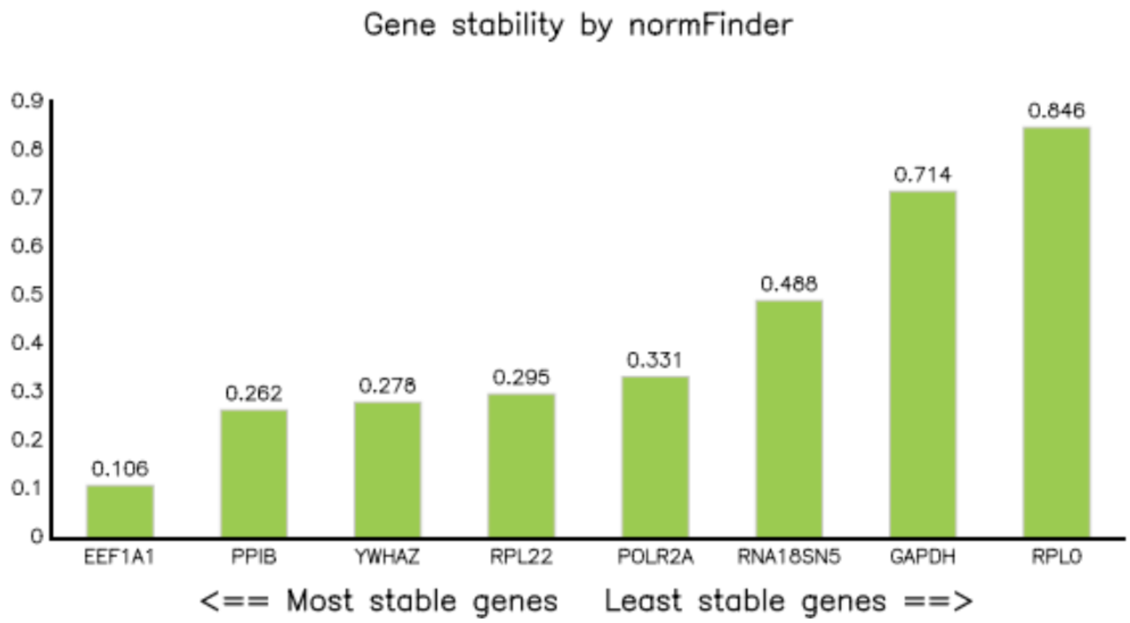

2.4 Genorm

Gene name	Stability value
PPIB | EEF1A1	0.293
YWHAZ	0.326
POLR2A	0.349
RPL22	0.411
RNA18SN5	0.456
GAPDH	0.538
RPL0	0.633

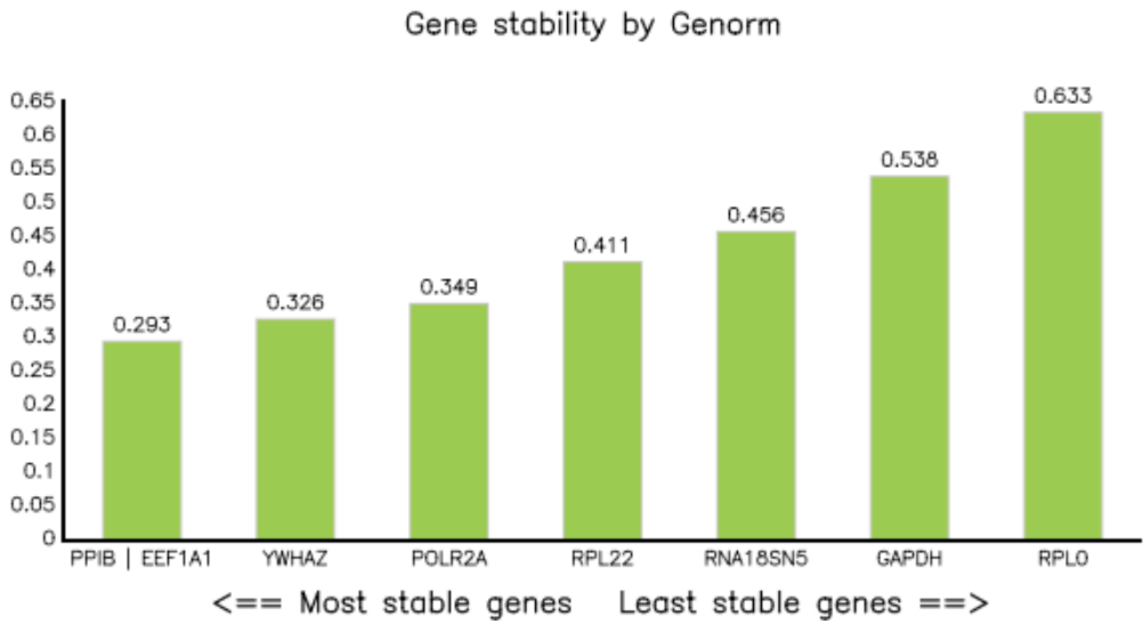

Supplement: Supplementary file 1 [file ijms-25-13525-s001.zip › Supplement_3_SH007_RefFinder.pdf]
