# Supplementary material for: Investigation of Oxidative-Stress Impact on Human Osteoblasts During Orthodontic Tooth Movement Using an In Vitro Tension Model
Source: Int J Mol Sci. 2024 Dec 17;25(24):13525. doi: 10.3390/ijms252413525 (PMC11677893; doi:10.3390/ijms252413525)
Supplement: Supplementary file 1 [file ijms-25-13525-s001.zip › ijms-3329106-graphic-issue-done+revised ref style.pdf]

Supplement 2 to manuscript

“Investigation of Oxidative-Stress Impact on Orthodontic Tooth Movement Using an In Vitro Tension Model”

Contents

Supplementary Table S2.1: MIQE checklist for the RT-qPCR workflow **2**

Supplementary Table S2.2: *In-silico* analysis of the RT-qPCR primer **5**

Supplementary Table S2.3: Primer validation by RT-qPCR **7**

References **8**

Supplementary Table S2.1: MIQE checklist for the RT-qPCR workflow

	Details	Checklist
**Sample/Template**		
Source	If cancer, was biopsy screened for adjacent normal tissue?	Human alveolar-bone derived osteoblasts (hOBs) were obtained anonymously from a male donor undergoing a surgical procedure based orthodontic treatment and isolated according to established procedures [1,2].
Method of preservation	Liquid N2/RNAlater/formalin	Cell lysates were snap frozen in liquid N2 and stored at -80°C until all samples were collected.
Storage time (if appropriate)	If using samples >6 months old	Not applicable.
Handling	Fresh/frozen/formalin	Cell lysates were prepared using RNA lysis buffer from Quick-RNA^{TM} MicroPrep kit (R1051; Zymo). They were snap-frozen in liquid nitrogen, and then stored at -80°C until further use for RNA extraction.
Extraction method	TriZol/columns	Defrosted cells lysates were passed through QIAshredder™ columns (Qiagen) to shear genomic DNA. The Quick-RNA™ Miniprep Kit (Zymo) was used for further RNA purification. After primary column purification, DNase I digestion was applied to reduce genomic DNA contamination as described by the manufacturer (Zymo). Finally, DNase/RNase-free water was used to elute the RNA from the columns. Before storage in the -80°C, RNase inhibitor RNasin® (Promega) was added to each preparation.
RNA:DNA-free	Intron-spanning primers/no RT control	Most primers were intron-spanning (Supplementary Table 2.2). Treatment with QIAshredder™ columns (Qiagen) and DNase I digestion were applied to reduce genomic DNA contamination according to the manufacturer's instructions (Zymo).
Concentration	Nanodrop/ribogreen/microfluidics	Purity and concentration of extracted RNA were detected photometrically (Nanodrop ND-1000; PeqLab). Ratio of $A_{260/280}$>1.8 was found, indicating free of protein contamination during RNA preparations.
RNA: integrity	Microfluidics/3':5' assay	No.
Inhibition-free	Method of testing	Serial dilution of cDNA as shown in “Primer efficiency” in Supplementary Table S2.3 below.
**Assay optimisation/validation**		
Accession number	RefSeq XX_1234567	Table 1 in the manuscript and Supplementary Table S2.2.

	Details	Checklist
Amplicon details	Exon location, amplicon size	Supplementary Table S2.2
Primer sequence	Even if previously published	Table 3 in the manuscript; Supplementary Tables S2.2 and S2.3.
*Probe sequence**	Identify LNA or other substitutions	No probes were used.
In silico	BLAST/Primer-BLAST/m-fold	Primer-BLAST, UCSC In-Silico PCR, and ENSEMBL were used for *in silico* testing.
empirical	Primer concentration/annealing temperature	The optimal annealing temperatures were first identified by gradient PCR (TProfessional Gradient; Biometra, Goettingen, Germany) and then were finalized by qPCR on Roche LightCycler® 480 (LC480). Optimal annealing temperatures are recorded in Supplementary Table S2.3 below.
Priming conditions	Oligo-dT/random/combination/target-specific	The SuperScript® IV First Strand Synthesis System (Invitrogen) was used for cDNA synthesis with random hexamers provided. For each cDNA synthesis reaction, 600 ng total RNA was used. Target-specific primers for qPCR were used after assessment (Table 1 of the manuscript; Supplementary Table S2.3).
PCR efficiency	Dilution curve	Information on serial dilutions and primer efficiency was summarized in Supplementary Table S2.3. For each gene, two technical replicates were used for each dilution for qPCR. For analysis of qPCR including the standard curves, LC480 software version 1.5.0.39 was used.
Linear dynamic range	Spanning unknown targets	The analyzing software for qPCR appointed the linear dynamic range automatically.
Limits of detection	LOD detection/accurate quantification	The analyzing software for qPCR appointed the LOD automatically.
Intra-assay variation	Copy numbers not Cq	Each gene was detected on one individual plate.
**RT/PCR**		
Protocols	Detailed description, concentrations, volumes	For real-time PCR, LightCycler® 480 SYBR Green I Master kit (04887352001; Roche Diagnostics GmbH, Mannheim, Germany) was used to detect gene expression of PTGS2/COX2, IL6, CXCL8/IL8, RUNX2, CASP3, CASP8, MAP1LC3A/LC3, BECN1, TNFRSF11B/OPG and P2RX7 using the LightCycler® 480 with LC480 software version 1.5.0.39 (both from Roche Molecular Diagnostics, Basel, Switzerland). According to the manufacturer’ protocol, 5 µl diluted cDNA (1:10 with double distilled, sterile water), 1 µl gene-specific forward primer, 1 µl gene-specific reverse primers, 3 µl PCR water and 10 µl qPCR mastermix were added for reaction. PCR reactions proceeded as follows: 10 min of initial denaturation at 95 °C and 45 cycles of amplifications. Each amplification consisted of three steps: 15 s of denaturation at 95 °C, 15 s of specific annealing temperature for each primer pair and 15 s of elongation at 72 °C.

	Details	Checklist
Reagents	Supplier, Lot number	Primers for genes were synthesized using sequences from related literatures. The primers were verified by *in silico* tests using related bioinformatic tools given in **Supplementary Table S2.2**. All primers were synthesized by TIB Molbiol Syntheselabor GmbH (Berlin, Germany). Information on the kits used (Quick-RNA™ MicroPrep kit; SuperScript® IV First Strand Synthesis kit, Invitrogen; LightCycler® 480 SYBR Green I Master kit, Roche) were all given in the manuscript.
Duplicate RT	ΔCq	No, but two technical replicates were repeated for each biological replicate at minimum.
NTC	Cq & melt curves	Yes
NAC	ΔCq beginning:end of qPCR	No, as no probes were used.
Positive control	Inter-run calibrators	No, each gene was tested on one plate with all samples included.
**Data analysis**		
Specialist software	e.g., QBAsePlus	IBM SPSS Statistics 29 (IBM Corp., Armonk, NY, USA)
Statistical justification	e.g., biological replicates	For each force magnitude for every force duration, three biological replicates were used. Each biological replicate was repeated with two technical replicates, giving a total of 6 amplifications of qPCR.
Transparent, validated normalization	e.g., GeNorm summary	After testing with RT-qPCR using cDNA from some samples and assessment with RefFinder, *EEF1A1* and *PPIB* were proved to be most stable in this experiment among the panel of reference genes. Therefore, *EEF1A1* and *PPIB* were used as reference genes for following analysis.

Supplementary Table S2.2: *In-silico* analysis of the RT-qPCR primer

**Official gene symbol**	**Reference sequence** (NCBI GenBank)	**5'-forward primer-3'** (length / T_m / %GC / Self-comp./Self-3'-comp.)	**5'-reverse primer-3'** (length / T_m / %GC / Self-comp./Self-3'-comp.)	**Amplicon length (bp)**	**Amplicon location** (bp of Start/Stop)	**Intron spanning** (length, bp)	**In silico qPCR specificity**	**Variants targeted** (Transcript/ Splice)	**Reference**
Genes of interest									
PTGS2	NM_000963.4	AAGCCTTCTCTAACCTCTCC (20 / 55.9°C / 50% / 5/0)	GCCCTCGCTTATGATCTGTC (20 / 58.2°C / 55% / 4/1)	234	510 / 743	Yes (430)	Yes (BLAST, UCSC)	Yes	[3], [4]
IL6	NM_000600.5	TGGCAGAAAACAACCTGAACC (21 / 56.5°C / 48% / 3/0)	TGGCTTGTTCCTCACTACTCTC (22 / 56.9°C / 50% / 2/0)	168	317 / 484	Yes (707)	Yes (BLAST, UCSC)	Yes	[3], [4]
CXCL8 / IL8	NM_000584.4	CAGAGACAGCAGAGCACACAA (21 / 60.5°C / 52% / 2/0)	TTAGCACTCCTTGGCAAAAC (20 / 56.5°C / 45% / 5/0)	170	10 / 179	Yes (819)	Yes (BLAST)	Yes	[5]
RUNX2	NM_001015051.4	GCGCATTCCTCATCCCAGTA (20 / 56.9°C / 55% / 4/2)	GGCTCAGGTAGGAGGGGTAA (20 / 56.9°C / 60% / 3/1)	176	947 / 1122	Yes (20131)	Yes (BLAST, UCSC)	Yes	[1], [3]
BECN1	NM_003766.5	AGGTTGAGAAAGGCGAGACA (20 / 58.9°C / 50% / 2/0)	AATTGTGAGGACACCCAAGC (20 / 58.3°C / 50% / 4/2)	196	1297 / 1492	Yes (726)	Yes (BLAST)	Yes	[6]
MAP1LC3A/LC3	NM_032514.4	CGTCCTGGACAAGACCAAGT (20 / 59.6°C / 55% / 7/2)	TCCTCGTCTTTCTCCTGCTC (20 / 58.8°C / 55% / 2/0)	183	286 / 468	Yes (179)	Yes (BLAST)	Yes	[6]
CASP3	NM_004346.4	TGGAGGCCGACTTCTTGTAT (20 / 58.4°C / 50% / 4/2)	ACTGTTTCAGCATGGCACAA (20 / 58.6°C / 45% / 4/2)	111	801 / 911	Yes (1535)	Yes (BLAST)	Yes	[7]
CASP8	NM_001228.5	GGAGGAGTTGTGTGGGGTAA (20 / 58.9°C / 55% / 2/1)	CCTGCATCCAAGTGTGTTCC (20 / 59.1°C / 55% / 4/0)	207	931 / 1137	Yes (1873)	Yes (BLAST)	Yes	[8]
TNFRSF11B	NM_002546.4	TCAAGCAGGAGTGCAATCG (19 / 54.9°C / 53% / 6/4)	AGAATGCCTCCTCACACAGG (20 / 56.3°C / 55% / 4/1)	342	342 / 683	Yes (6020)	Yes (BLAST, UCSC)	Yes	[9]
P2RX7	NM_002562.6	AGTGCGAGTCCATTGTGGAG (20 / 57.0°C / 55% / 5/3)	CATCGCAGGTCTTGGGACTT (20 / 57.0°C / 55% / 5/2)	143	1303 / 1445	Intron- and exon-spanning	Yes (BLAST, UCSC)	Yes	[1]
Reference genes									
RPL0	NM_001002.4	GAAACTCTGCATTCTCGCTTCC (22 / 57.4°C / 50% / 4/0)	GACTCGTTTGTACCCGTTGATG (22 / 57.1°C / 50% / 4/0)	120	702 / 821	Yes (1091)	Yes (BLAST/UCSC)	Yes	[10], [11]
RPL22	NM_000983.4	TGATTGCACCCACCCTGTAG (20 / 56.6°C / 55% / 4/2)	GGTTCCCAGCTTTTCCGTTC (20 / 56.4°C / 55% / 4/0)	98	91 / 188	Yes (4597)	Yes (BLAST/UCSC)	Yes	[10], [11]
GAPDH	NM_002046.7	CTCCTGTTCGACAGTCAGCC (20 / 57.4°C / 60% / 6/1)	CGACCAAATCCGTTGACTCC (20 / 55.9°C/ 55%/ 3/1)	103	12 / 114	Yes, rev. primer on exon junction	Yes (BLAST/UCSC)	Yes	[10], [12]
EEF1A1	NM_001402.6	CCTGCCTCTCCAGGATGTCTAC (22 / 59.0°C / 59% / 5/2)	GGAGCAAAGGTGACCACCATAC (22 / 58.7°C / 55% / 6/2)	105	804 / 908	Yes (87)	Yes (BLAST/UCSC)	Yes	[10], [11]
PPIB	NM_000942.5	TTCCATCGTGTAATCAAGGACTTC (24 / 56.7°C / 42% / 4/2)	GCTCACCGTAGATGCTCTTTC (21 / 56.1°C / 52% / 4/0)	88	313 / 400	Yes (3194)	Yes (BLAST/UCSC)	Yes	[10], [11]
YWHAZ	NM_003406.4	AGGAGATTACTACCGTTACTTGGC (24 / 57.8°C / 46% / 4/2)	AGCTTCTTGGTATGCTTGTTGTG (23 / 57.4°C / 43% / 4/0)	91	491 / 581	Yes (617)	Yes (BLAST/UCSC)	Yes	[10], [11]
RNA18SN5	NR_003286.4	AACTGCGAATGGCTCATTAAATC (23 / 55.8°C / 39% / 6/3)	GCCCGTCGGCATGTATTAG (19 / 55.2°C / 58% / 5/1)	103	84 / 186	No (rRNA)	No (RNA45S5 also targeted)	-	[10], [11]
POLR2A	NM_000937.5	TCGCTTACTGTCTTCCTGTTGG (22 / 57.8°C / 50% / 3/0)	TGTGTTGGCAGTCACCTTCC (20 / 57.4°C / 55% / 3/3)	108	3811 / 3918	Yes (468)	Yes (BLAST/UCSC)	Yes	[10], [11]

T_m, melting temperature of primer or qPCR product (amplicon); %GC, percent of guanin/cytosine content; bp, base pairs; Self-comp., self-complementary; Self-3'-comp., self 3' complementary.

To perform silico analysis of RT-qPCR primers, their targets and corresponding amplification products, the following programs and online resources were used. All URLs were valid on 02-12-2020.

a. Primer-BLAST (URL: https://www.ncbi.nlm.nih.gov/tools/primer-blast/) was used to check the melting temperature (T_m) and the “length” of each primer, their “in silico qPCR specificity”, possible co-amplification of genomic DNA, “Self-comp.” and “Self-3'-comp”.
    i. Settings for “Refseq mRNA”: max primer annealing: 65; intron length range: 100 – 100000, max. target amplicon size: 40000
    ii. Settings for “Refseq representative genomes”: max primer annealing: 65; intron length range: 100 – 100000, max. target amplicon size: 40000
b. UCSC In-Silico PCR (URL: https://genome.ucsc.edu/cgi-bin/hgPcr) was used to check “In silico qPCR specificity” and RT-qPCR in genomic context.
c. “Amplicon (length)”, “Amplicon location (bp of Start/Stop)”, “Intron-spanning (length)” was identified or calculated by either Primer-BLAST or UCSC In-Silico PCR
d. ENSEMBL (URL: https://www.ensembl.org) was used to check “Variants targeted (Transcript/Splice)”.

Supplementary Table S2.3: Primer validation by RT-qPCR

Gene symbol	Primer sequence (f: 5'-forward primer-3'; r: 5'-reverse primer-3')	Reference	Specificity by melting curve / T_m (°C)	Specificity by agarose gel / amplicon size (bp)	Annealing temp. (°C)	Dilution series used for efficiency testing – starting from 1:10 prediluted cDNA	Primer efficiency			
							**Efficiency**	**Error**	**Slope**	**Y intercept**
Genes of interest										
PTGS2	f: AAGCCTTCTCTAACCTCTCC<br>r: GCCCTCGCTTATGATCTGTC	[3], [4]	Yes / 81.7	234	58	1:1, 1:10, 1:100, 1:1000	2.028	0.0768	-3.257	35.67
IL6	f: TGGCAGAAAACAACCTGAACC<br>r: TGGCTTGTTCCTCACTACTCTC	[3], [4]	Yes / 80.3	168	58	1:1, 1:10, 1:100, 1:1000	1.999	0.0277	-3.325	39.05
CXCL8	f: CAGAGACAGCAGAGCACACAA<br>r: TTAGCACTCCTTGGCAAAAC	[5]	Yes/ 84.5	170	55	1:1, 1:4, 1:16, 1:64, 1:256	2.082	0.025	-3.141	34.17
RUNX2	f: GCGCATTCCTCATCCCAGTA<br>r: GGCTCAGGTAGGAGGGGTAA	[1], [3]	Yes / 85.3	176	58	1:1, 1:10, 1:100, 1:1000	1.94	0.00746	-3.475	39.04
BCN1	f: AGGTTGAGAAAGGCGAGACA<br>r: AATTGTGAGGACACCCAAGC	[6]	Yes/ 82.7	196	58	1:1, 1:10, 1:100, 1:1000	2.037	0.0676	-3.237	33.41
MAP1LC3A	f: CGTCCTGGACAAGACCAAGT<br>r: TCCTCGTCTTTCTCCTGCTC	[6]	Yes/ 87.8	183	58	1:1, 1:10, 1:100, 1:1000	1.951	0.0674	-3.445	34.7
CASP3	f: TGGAGGCCGACTTCTTGTAT<br>r: ACTGTTTCAGCATGGCACAA	[7]	Yes/ 81.7	111	58	1:1, 1:10, 1:100, 1:1000	1.984	0.01	-3.362	37.23
CASP8	f: GGAGGAGTTGTGTGGGGTAA<br>r: CCTGCATCCAAGTGTGTTCC	[8]	Yes/ 81.0	207	58	1:1, 1:10, 1:100, 1:1000	2.033	0.0614	-3.245	35.23
TNFRSF11B	f: TCAAGCAGGAGTGCAATCG<br>r: AGAATGCCTCCTCACACAGG	[9]	Yes / 84.8	342	64	1:1, 1:10, 1:100, 1:1000, 1:10000	1.915	0.0164	-3.545	37.27
P2RX7	f: AGT GCG AGT CCA TTG TGG AG<br>r: CAT CGC AGG TCT TGG GAC TT	[1]	Yes/ 82.5	143	58	undiluted, 1:2, 1:4, 1:8	2.048	0.0298	-3.211	35.24
Reference genes										
RPL0	f: GAAACTCTGCATTCTCGCTTCC<br>r: GACTCGTTTGTACCCGTTGATG	[10], [11]	Yes / 83.8	120	64	1:1, 1:10, 1:100, 1:1000, 1:10000	1.947	0.027	-3.455	32.54
RPL22	f: TGATTGCACCCACCCTGTAG<br>r: GGTTCCCAGCTTTTCCGTTC	[10], [11]	Yes / 80.1	98	61	1:1, 1:10, 1:100, 1:1000, 1:10000	2.004	0.007	-3.313	33.4
GAPDH	f: CAACTACATGGTTTACATGTTC<br>r: GCCAGTGGACTCCACGAC	[10], [12]	Yes / 84.4	103	52	1:1, 1:10, 1:100, 1:1000, 1:10000	1.968	0.002	-3.4	30.4
EEF1A1	f: CCTGCCTCTCCAGGATGTCTAC<br>r: GGAGCAAAGGTGACCACCATAC	[10], [11]	Yes / 82.4	105	61	1:1, 1:10, 1:100, 1:1000, 1:10000	2.038	0.002	-3.235	29.41
PPIB	f: TTCCATCGTGTAATCAAGGACTTC<br>r: GCTCACCGTAGATGCTCTTTC	[10], [11]	Yes / 82.4	88	55	1:1, 1:10, 1:100, 1:1000, 1:10000	1.992	0.007	-3.341	33.77
YWHAZ	f: AGGAGATTACTACCGTTACTTGGC<br>r: AGCTTCTTGGTATGCTTGTTGTG	[10], [11]	Yes / 81.3	91	55	1:1, 1:10, 1:100, 1:1000, 1:10000	2.019	0.012	-3.276	33.48
RNA18SN5	f: AACTGCGAATGGCTCATTAAATC<br>r: GCCCGTCGGCATGTATTAG	[10], [11]	Yes / 80.4	103	55	1:1, 1:10, 1:100, 1:1000, 1:10000	2.022	0.021	-3.271	19.26
POLR2A	f: TCGCTTACTGTCTTCCTGTTGG<br>r: TGTGTTGGCAGTCACCTTCC	[10], [11]	Yes / 83.7	108	58	1:1, 1:10, 1:100, 1:1000, 1:10000	2.046	0.016	-3.216	34.72

References

1. Shi, J.; Folwaczny, M.; Wichelhaus, A.; Baumert, U. Differences in *RUNX2* and *P2RX7* gene expression between mono- and coculture of human periodontal ligament cells and human osteoblasts under compressive force application. *Orthod. Craniofac. Res.* **2019**, *22*, 168-176.
2. Ng, K.W.; Schantz, J.-T. *A Manual for Primary Human Cell Culture*, 2nd ed.; World Scientific: Hackensack, NJ, USA, 2010; pp. 38-47.
3. Janjic Rankovic, M.; Docheva, D.; Wichelhaus, A.; Baumert, U. Effect of static compressive force on in vitro cultured PDL fibroblasts: monitoring of viability and gene expression over 6 days. *Clin. Oral Investig.* **2020**, *24*, 2497-2511.
4. Shi, J.; Baumert, U.; Folwaczny, M.; Wichelhaus, A. Influence of static forces on the expression of selected parameters of inflammation in periodontal ligament cells and alveolar bone cells in a co-culture in vitro model. *Clin. Oral Investig.* **2019**, *23*, 2617-2628.
5. Jones, R.L.; Hannan, N.J.; Kaitu'u, T.J.; Zhang, J.; Salamonsen, L.A. Identification of chemokines important for leukocyte recruitment to the human endometrium at the times of embryo implantation and menstruation. *J. Clin. Endocrinol. Metab.* **2004**, *89*, 6155-6167.
6. Zhuang, H.; Hu, D.; Singer, D.; Walker, J.V.; Nisr, R.B.; Tieu, K.; Ali, K.; Tredwin, C.; Luo, S.; Ardu, S.; et al. Local anesthetics induce autophagy in young permanent tooth pulp cells. *Cell Death Discov.* **2015**, *1*, 15024.
7. Wang, Y.; Du, C.; Wan, W.; He, C.; Wu, S.; Wang, T.; Wang, F.; Zou, R. shRNA knockdown of integrin-linked kinase on hPDLCs migration, proliferation, and apoptosis under cyclic tensile stress. *Oral Dis.* **2020**, *26*, 1747-1754.
8. Cao, Z.; Zhang, H.; Cai, X.; Fang, W.; Chai, D.; Wen, Y.; Chen, H.; Chu, F.; Zhang, Y. Luteolin Promotes Cell Apoptosis by Inducing Autophagy in Hepatocellular Carcinoma. *Cell. Physiol. Biochem.* **2017**, *43*, 1803-1812.
9. Yang, Y.; Yang, Y.; Li, X.; Cui, L.; Fu, M.; Rabie, A.B.; Zhang, D. Functional analysis of core binding factor a1 and its relationship with related genes expressed by human periodontal ligament cells exposed to mechanical stress. *Eur. J. Orthod.* **2010**, *32*, 698-705.
10. Sun, C.; Janjic Rankovic, M.; Folwaczny, M.; Stocker, T.; Otto, S.; Wichelhaus, A.; Baumert, U. Effect of Different Parameters of In Vitro Static Tensile Strain on Human Periodontal Ligament Cells Simulating the Tension Side of Orthodontic Tooth Movement. *Int. J. Mol. Sci.* **2022**, *23*, 1525.
11. Nazet, U.; Schröder, A.; Spanier, G.; Wolf, M.; Proff, P.; Kirschneck, C. Simplified method for applying static isotropic tensile strain in cell culture experiments with identification of valid RT-qPCR reference genes for PDL fibroblasts. *Eur. J. Orthod.* **2020**, *42*, 359-370.
12. Chirieleison, S.M.; Marsh, R.A.; Kumar, P.; Rathkey, J.K.; Dubyak, G.R.; Abbott, D.W. Nucleotide-binding oligomerization domain (NOD) signaling defects and cell death susceptibility cannot be uncoupled in X-linked inhibitor of apoptosis (XIAP)-driven inflammatory disease. *J. Biol. Chem.* **2017**, *292*, 9666-9679.
