# Supplementary material for: Investigation of Oxidative-Stress Impact on Human Osteoblasts During Orthodontic Tooth Movement Using an In Vitro Tension Model
Source: Int J Mol Sci. 2024 Dec 17;25(24):13525. doi: 10.3390/ijms252413525 (PMC11677893; doi:10.3390/ijms252413525)
Supplement: Supplementary file 1 [file ijms-25-13525-s001.zip › Supplement_1_SH007_tension10_results.pdf]

Supplement 1 to manuscript

“Investigation of Oxidative-Stress Impact on Orthodontic Tooth Movement Using an In Vitro Tension Model”

Content

Influence of tension during recovery **2**

Results from RT-qPCR *2*

Graphic - Inflammation *4*

Graphic - Bone remodelling *5*

Graphic - Autophagy and apoptosis *5*

Live/Dead cell staining **6**

Influence of tension during recovery

Results from RT-qPCR

The adjusted *p*-values are reported.

Analyte	Treatment	N	Mean	SD	Median	Min	Max	K-W	Vs. ctrl.		Adj. multiple comparisons
IL8_dCq	Ctrl	6	8.56	0.04	8.56	8.48	8.61	0.003		**	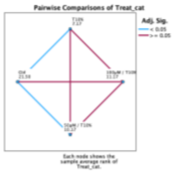
	T10%	6	7.28	0.42	7.29	6.75	7.74		0.003	**	
	50µM / T10%	6	7.68	0.52	7.56	6.98	8.47		0.033	*	
	100µM / T10%	6	7.71	0.38	7.68	7.22	8.38		0.068	n.s.	
IL8_FC	Ctrl	6	1.00	0.03	1.00	0.96	1.05				
	T10%	6	2.52	0.73	2.40	1.76	3.49				
	50µM / T10%	6	1.93	0.67	2.00	1.06	2.98				
	100µM / T10%	6	1.84	0.44	1.83	1.13	2.52				
IL6_dCq	Ctrl	6	10.43	0.07	10.43	10.33	10.55	<0.001		***	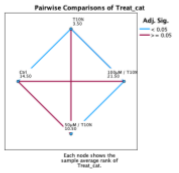
	T10%	6	9.15	0.12	9.15	9.00	9.31		0.042	*	
	50µM / T10%	6	10.26	0.17	10.32	10.03	10.43		1.000	n.s.	
	100µM / T10%	6	11.21	0.16	11.13	11.09	11.42		0.516	n.s.	
IL6_FC	Ctrl	6	1.00	0.05	1.00	0.92	1.07				
	T10%	6	2.44	0.21	2.43	2.17	2.69				
	50µM / T10%	6	1.13	0.14	1.08	1.00	1.32				
	100µM / T10%	6	0.59	0.06	0.62	0.50	0.63				
COX2_dCq	Ctrl	6	6.64	0.06	6.64	6.54	6.74	<0.001		***	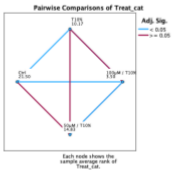
	T10%	6	5.89	0.12	5.89	5.73	6.03		0.033	*	
	50µM / T10%	6	6.07	0.10	6.06	5.95	6.24		0.613	n.s.	
	100µM / T10%	6	4.66	0.11	4.66	4.52	4.81		<0.001	***	
COX2_FC	Ctrl	6	1.00	0.04	1.00	0.93	1.07				
	T10%	6	1.69	0.14	1.68	1.53	1.88				
	50µM / T10%	6	1.49	0.10	1.50	1.32	1.62				
	100µM / T10%	6	3.98	0.30	3.97	3.56	4.35				
RUNX2_dCq	Ctrl	6	6.72	0.13	6.71	6.61	6.97	<0.001		***	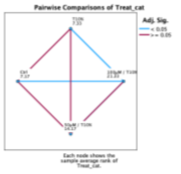
	T10%	6	6.64	0.43	6.60	6.04	7.20		1.000	n.s.	
	50µM / T10%	6	7.23	0.41	7.12	6.71	7.89		0.518	n.s.	
	100µM / T10%	6	8.05	0.12	8.05	7.86	8.20		0.003	**	
RUNX2_FC	Ctrl	6	1.00	0.09	1.01	0.84	1.08				
	T10%	6	1.10	0.33	1.10	0.72	1.60				
	50µM / T10%	6	0.73	0.20	0.76	0.45	1.01				
	100µM / T10%	6	0.40	0.03	0.40	0.36	0.45				

Analyte	Treatment	N	Mean	SD	Median	Min	Max	K-W	Vs. ctrl.		Adj. multiple comparisons
CASP3_dCq	Ctrl	6	6.41	0.01	6.41	6.39	6.43	<0.001		***	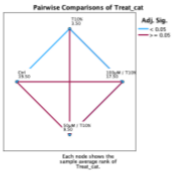
	T10%	6	4.72	0.09	4.72	4.59	4.85		0.001	**	
	50µM / T10%	6	5.07	0.04	5.07	5.03	5.14		0.084	n.s.	
	100µM / T10%	6	6.22	0.54	6.22	5.58	6.88		1.000	n.s.	
CASP3_FC	Ctrl	6	1.00	0.01	1.00	0.99	1.01				
	T10%	6	3.24	0.20	3.24	2.95	3.53				
	50µM / T10%	6	2.53	0.07	2.53	2.41	2.60				
	100µM / T10%	6	1.21	0.44	1.14	0.72	1.78				
CASP8_dCq	Ctrl	6	7.61	0.02	7.61	7.59	7.64	0.001		**	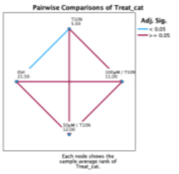
	T10%	6	6.71	0.33	6.53	6.43	7.17		0.001	**	
	50µM / T10%	6	7.20	0.32	7.24	6.68	7.56		0.119	n.s.	
	100µM / T10%	6	7.05	0.33	7.15	6.61	7.49		0.060	n.s.	
CASP8_FC	Ctrl	6	1.00	0.01	1.00	0.98	1.01				
	T10%	6	1.91	0.40	2.11	1.36	2.27				
	50µM / T10%	6	1.35	0.32	1.30	1.04	1.91				
	100µM / T10%	6	1.51	0.36	1.38	1.09	2.00				
LC3_dCq	Ctrl	6	7.88	0.17	7.88	7.63	8.07	<0.001		***	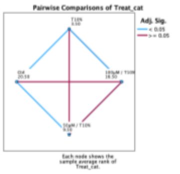
	T10%	6	6.63	0.15	6.63	6.46	6.84		<0.001	***	
	50µM / T10%	6	7.12	0.12	7.12	6.96	7.27		0.042	*	
	100µM / T10%	6	7.66	0.18	7.64	7.44	7.98		1.000	n.s.	
LC3_FC	Ctrl	6	1.01	0.12	1.00	0.88	1.19				
	T10%	6	2.39	0.24	2.38	2.06	2.68				
	50µM / T10%	6	1.70	0.14	1.69	1.53	1.89				
	100µM / T10%	6	1.17	0.14	1.18	0.93	1.36				
BECN1_dCq	Ctrl	6	6.80	0.03	6.79	6.76	6.86	<0.001		***	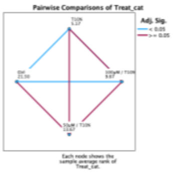
	T10%	6	5.12	0.06	5.12	5.04	5.20		<0.001	***	
	50µM / T10%	6	5.52	0.28	5.60	5.12	5.83		0.328	n.s.	
	100µM / T10%	6	5.27	0.21	5.27	5.07	5.65		0.022	*	
BECN1_FC	Ctrl	6	1.00	0.02	1.00	0.96	1.02				
	T10%	6	3.21	0.13	3.20	3.02	3.38				
	50µM / T10%	6	2.46	0.49	2.30	1.95	3.19				
	100µM / T10%	6	2.90	0.39	2.88	2.21	3.31				
OPG_dCq	Ctrl	6	3.13	0.03	3.13	3.09	3.17	<0.001		***	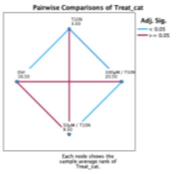
	T10%	6	2.16	0.23	2.12	1.84	2.50		0.009	**	
	50µM / T10%	6	2.70	0.12	2.67	2.57	2.87		0.518	n.s.	
	100µM / T10%	6	3.29	0.14	3.32	3.04	3.46		1.000	n.s.	
OPG_FC	Ctrl	6	1.00	0.02	1.00	0.97	1.03				
	T10%	6	1.98	0.31	2.02	1.54	2.44				
	50µM / T10%	6	1.35	0.11	1.37	1.20	1.47				

Analyte	Treatment	N	Mean	SD	Median	Min	Max	K-W	Vs. ctrl.		Adj. multiple comparisons
	100µM / T10%	6	0.90	0.09	0.88	0.79	1.06				
P2RX7_dCq	Ctrl	6	10.19	0.04	10.19	10.14	10.25	<0.001		***	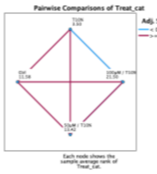
	T10%	6	9.15	0.12	9.15	9.00	9.31		0.285	n.s.	
	50µM / T10%	6	10.23	0.16	10.27	10.03	10.43		1.000	n.s.	
	100µM / T10%	6	11.19	0.13	11.13	11.09	11.41		0.090	n.s.	
P2RX7_FC	Ctrl	6	1.00	0.03	1.00	0.96	1.03				
	T10%	6	2.05	0.18	2.05	1.83	2.27				
	50µM / T10%	6	0.97	0.11	0.95	0.84	1.11				
	100µM / T10%	6	0.50	0.04	0.52	0.43	0.53				
PGE2ELISA	Ctrl	6	91.91	6.85	93.31	82.88	100.73	<0.001		***	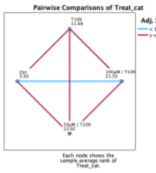
	T10%	6	130.92	3.85	130.97	125.15	135.78		0.379	n.s.	
	50µM / T10%	6	135.73	6.51	136.09	125.15	143.78		0.064	n.s.	
	100µM / T10%	6	200.03	13.56	202.74	182.44	219.06		<0.001	***	
IL6ELISA	Ctrl	6	230.68	16.07	236.51	205.93	245.50	0.006		**	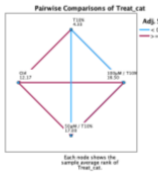
	T10%	6	208.60	2.83	208.24	205.04	213.70		0.330	n.s.	
	50µM / T10%	6	242.60	14.94	243.72	225.49	262.82		1.000	n.s.	
	100µM / T10%	6	241.29	6.99	243.25	232.04	248.81		1.000	n.s.	

Graphic - Inflammation

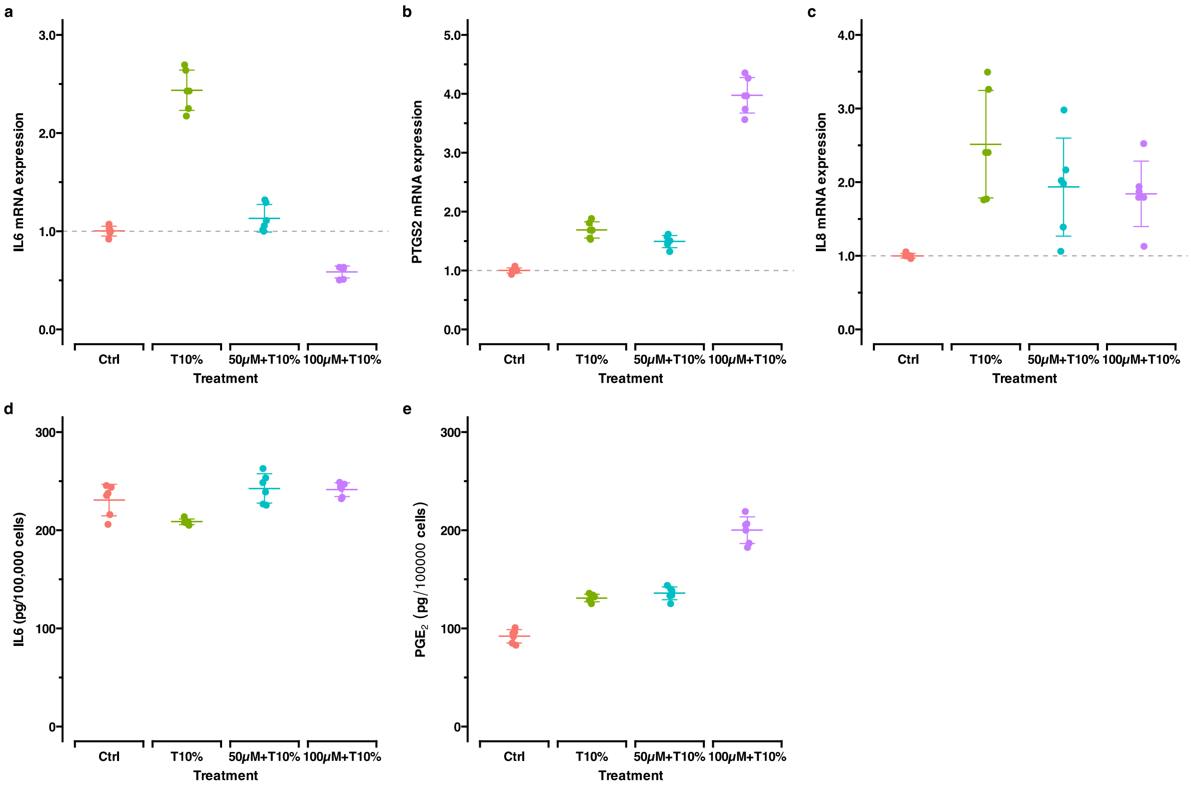

Graphic - Bone remodeling

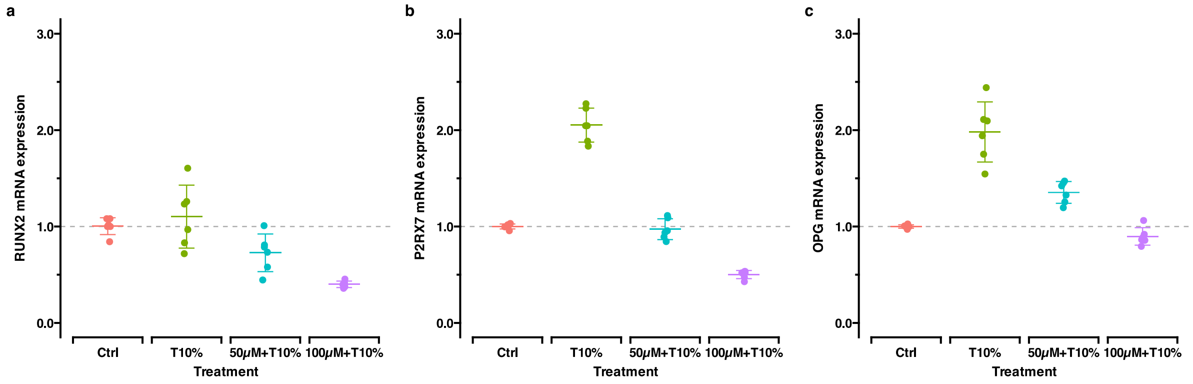

Graphic - Autophagy and apoptosis

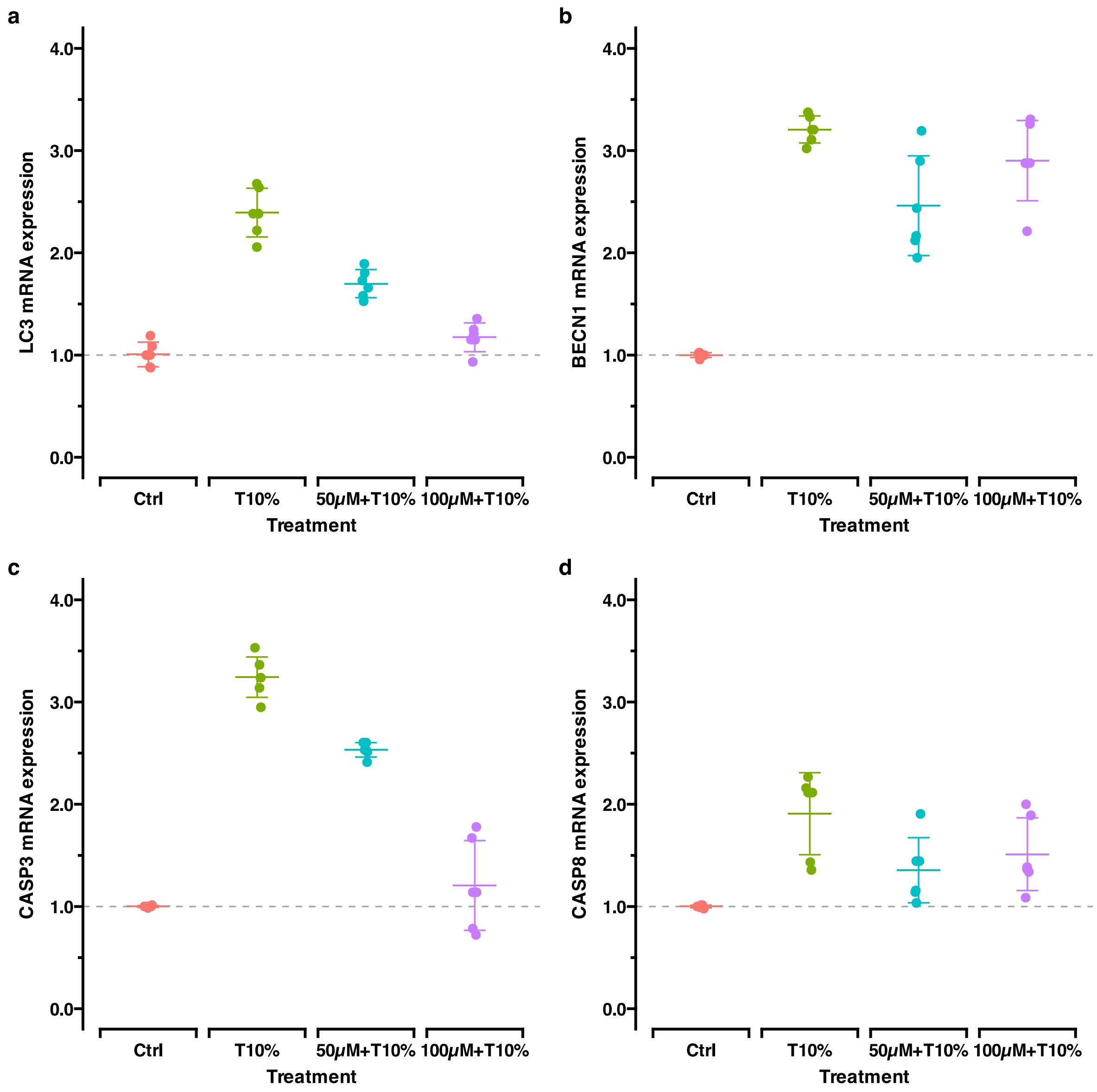

Live/Dead cell staining

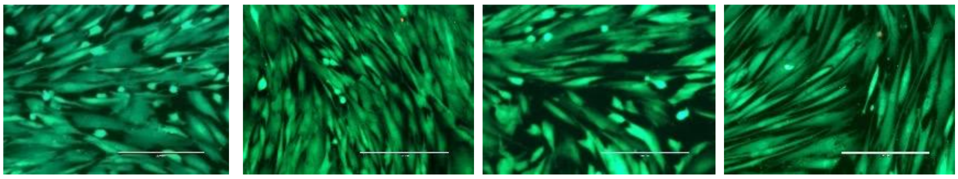

H_2O_2 (μM)	0	0	50	100
Tension (%)	0	10	10	10
